# m6A-methylated KCTD21-AS1 regulates macrophage phagocytosis through CD47 and cell autophagy through TIPR

**DOI:** 10.1038/s42003-024-05854-x

**Published:** 2024-02-21

**Authors:** Dong-Min Liang, You-Jie Li, Jia-Xiang Zhang, Huan-Huan Shen, Chun-Xia Wu, Ning Xie, Yan Liang, Yan-Mei Li, Jiang-Nan Xue, Hong-Fang Sun, Qin Wang, Jian Yang, Xiao-Hua Li, Ping-Yu Wang, Shu-Yang Xie

**Affiliations:** 1 Department of Biochemistry and Molecular Biology, Binzhou Medical University, YanTai, Shandong 264003 PR China; 2 Shandong Laboratory of Advanced Materials and Green Manufacturing (Yantai), Shandong, 264000 PR China; 3 Department of Chest Surgery, Yantaishan Hospital, Yantai, Shandong 264000 PR China; 4 Department of Immune Rheumatism, Yantaishan Hospital, Yantai, Shandong 264000 PR China; 5 Department of Immunology, Binzhou Medical University, Yantai, Shandong 264003 PR China; 6 Yantai Central Blood Station, Yantai, Shandong 264003 PR China; 7 Department of Epidemiology, Binzhou Medical University, YanTai, ShanDong 264003 PR China

**Keywords:** Targeted therapies, Non-small-cell lung cancer

## Abstract

Blocking immune checkpoint CD47/SIRPα is a useful strategy to engineer macrophages for cancer immunotherapy. However, the roles of CD47-related noncoding RNA in regulating macrophage phagocytosis for lung cancer therapy remain unclear. This study aims to investigate the effects of long noncoding RNA (lncRNA) on the phagocytosis of macrophage via CD47 and the proliferation of non-small cell lung cancer (NSCLC) via TIPRL. Our results demonstrate that lncRNA KCTD21-AS1 increases in NSCLC tissues and is associated with poor survival of patients. KCTD21-AS1 and its m6A modification by Mettl14 promote NSCLC cell proliferation. miR-519d-5p gain suppresses the proliferation and metastasis of NSCLC cells by regulating CD47 and TIPRL. Through ceRNA with miR-519d-5p, KCTD21-AS1 regulates the expression of CD47 and TIPRL, which further regulates macrophage phagocytosis and cancer cell autophagy. Low miR-519d-5p in patients with NSCLC corresponds with poor survival. High TIPRL or CD47 levels in patients with NSCLC corresponds with poor survival. In conclusion, we demonstrate that KCTD21-AS1 and its m6A modification promote NSCLC cell proliferation, whereas miR-519d-5p inhibits this process by regulating CD47 and TIPRL expression, which further affects macrophage phagocytosis and cell autophagy. This study provides a strategy through miR-519-5p gain or KCTD21-AS1 depletion for NSCLC therapy by regulating CD47 and TIPRL.

## Abstract (web summary)

The m6A-methylated form of the lncRNA KCTD21-AS1 influences CD47 and TIPRL expression via miR-519d-5p, impacting macrophage phagocytosis and autophagy in non-small cell lung cancer (NSCLC) tissues and ultimately correlating with poor survival in NSCLC patients.

## Introduction

The tumor microenvironment (TME) comprises tumor cells, as well as extracellular matrix, stromal cells, and immune cells. Immune cells within the TME include tumor-associated macrophages (TAMs), regulatory T cells, cytotoxic CD8 T cells, and natural killer cells^1^, which affect cancer progression and clinical therapy^2^. The TME also contains multiple extracellular soluble molecules, such as cytokines, chemotactic factor, and growth factors^1^. Thus, understanding the biology of the TME provides novel attractive strategies to regulate tumor proliferation or metastasis by blocking the above mentioned particular components.

TAMs play crucial roles in the majority of human solid malignancies^3,4^. TAM heterogeneity has been revealed within and across tumors, or across different cancer patients and across different lesions of the same patient^4,5^. Part of TAM heterogeneity, which may originate from a pro-inflammatory (so-called M1-like) to an anti-inflammatory (so-called M2-like) state, accounts for tumor environmental changes and affects tumorigenesis, vascularization, immunity, and resistance to drug therapy^6^.

CD47, as an antiphagocytic “don’t eat me” signal, is important for cancer treatment^7,8^. By combining with its receptor, signal regulatory protein alpha (SIRPα), on macrophages, CD47 passes a negative signaling cascade in the macrophages to suppress phagocytosis^9^. Therefore, CD47 blockade promotes the phagocytic ability of macrophages in the TME and contributes to tumor immunotherapy.

Different tumors accompany epigenetic alterations, as a different expression of noncoding RNA, to affect the gene expression and stability of the TME^10^. miR-200a can affect nasopharyngeal carcinoma cell proliferation, migration, and invasion by regulating CD47, thereby providing a potential form of cancer therapy^11^. miR-128 can also inhibit cancer cell proliferation, clonogenicity, and invasion, as well as promote phagocytosis in macrophages via CD47^12^. However, little is known about the role of CD47-related noncoding RNA in regulating the phagocytosis of macrophage for lung cancer therapy. In this study, we investigated the role of CD47-related noncoding RNA in the tumorigenesis of lung cancer. Results demonstrated that KCTD21-AS1 inhibited the phagocytic function of macrophages through CD47 and promoted the tumorigenesis of NSCLC.

## Results

### KCTD21-AS1 increased in NSCLC tissues, associated with poor survival in patients

**Fig. 1:**
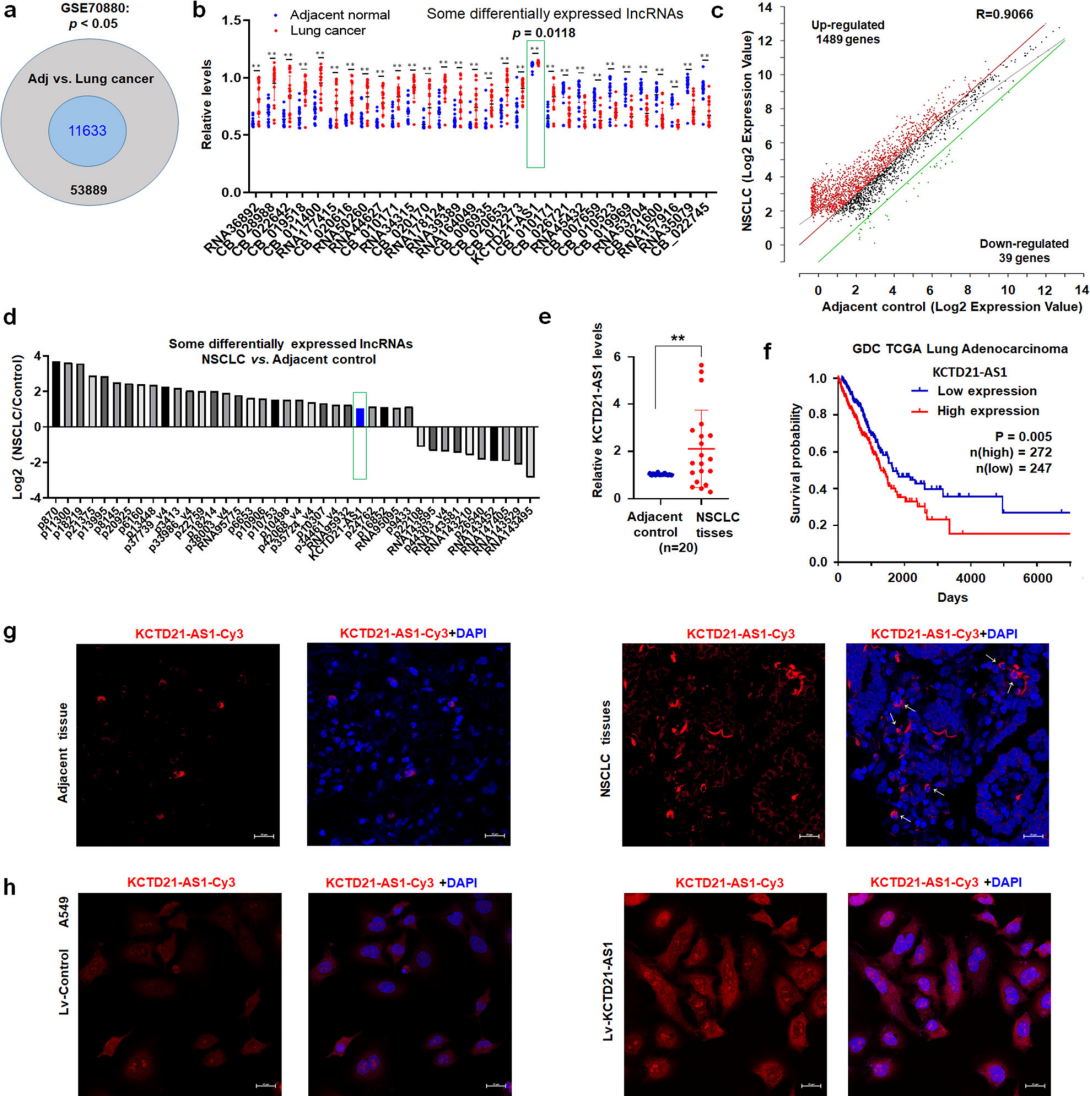
Expression of KCTD21-AS1 increased in lung cancer. **a** Number of different lncRNA expression levels. **b** Different expression levels of some lncRNAs including KCTD21-AS1 between lung cancer and adjacent normal tissues from GSE70880. **p < 0.01, Mann–Whitney U test. Data were shown as median (interquartile range). **c** Results of lncRNA microarray showed the up-regulated or down-regulated genes between lung adenocarcinoma and adjacent controls. **d** Different expression levels of some lncRNAs from lncRNA microarray, including KCTD21-AS1. **e** Levels of lncRNA were detected by qRT-PCR in lung adenocarcinoma tissues and adjacent normal tissues (N = 20). Data were shown as median (interquartile range), **p < 0.01, Mann–Whitney U test. **f** Overall survival analysis of patients with lung adenocarcinoma. **g** In situ hybridization showed that KCTD21-AS1 expression in lung adenocarcinoma tissues. Red indicates the expression of KCTD21-AS1. Bar = 20 μm. **h** In situ hybridization detection of KCTD21-AS1 in A549 cells. Red indicates the expression of KCTD21-AS1. Bar = 20 μm.

LncRNAs participate in immunotherapy response and TME, thereby affecting the tumorigenesis and progression of lung cancer^13^. Here, the GSE70880 dataset from GEO (http://www.ncbi.nlm.nih.gov/geo/) was downloaded to analyze the expression of lncRNAs in lung cancer. A total of 11,633 lncRNAs, including KCTD21-AS1, were higher in lung cancer tissues than in controls (Fig. 1a, b; Supplementary Fig. 1a). Data from TCGA also showed that KCTD21-AS1 levels increased in NSCLC tissues, including lung adenocarcinoma (n = 533, *p* < 0.01) and squamous cell carcinoma (n = 502, *p* < 0.01) tissues than in normal tissues (Supplementary Fig. 1b–d) through UALCAN analysis (http://ualcan.path.uab.edu/).

The main histological type of NSCLC is lung adenocarcinoma. Therefore, RNA was isolated from lung adenocarcinoma tissues and controls and analyzed by lncRNA array and qRT-PCR. The results further supported that KCTD21-AS1 levels were higher in lung adenocarcinoma tissues than in the control (Fig. 1c–e). Then, we analyzed the effect of KCTD21-AS1 expression on the overall survival of patients with lung adenocarcinoma through the Kaplan–Meier plotter (data from GDC TCGA Lung Adenocarcinoma, https://xenabrowser.net/). The results indicated that high KCTD21-AS1 levels in patients with lung adenocarcinoma were related to poor survival compared with low levels (*p* < 0.05; Fig. 1f). Results of in situ hybridization showed that KCTD21-AS1 expression increased in NSCLC tissues compared with controls (Fig. 1g). These results indicated that KCTD21-AS1 may play an oncogenic role in the tumorigenesis of NSCLC.

### KCTD21-AS1, m6A modification by Mettl14, and promoted NSCLC cell proliferation

**Fig. 2:**
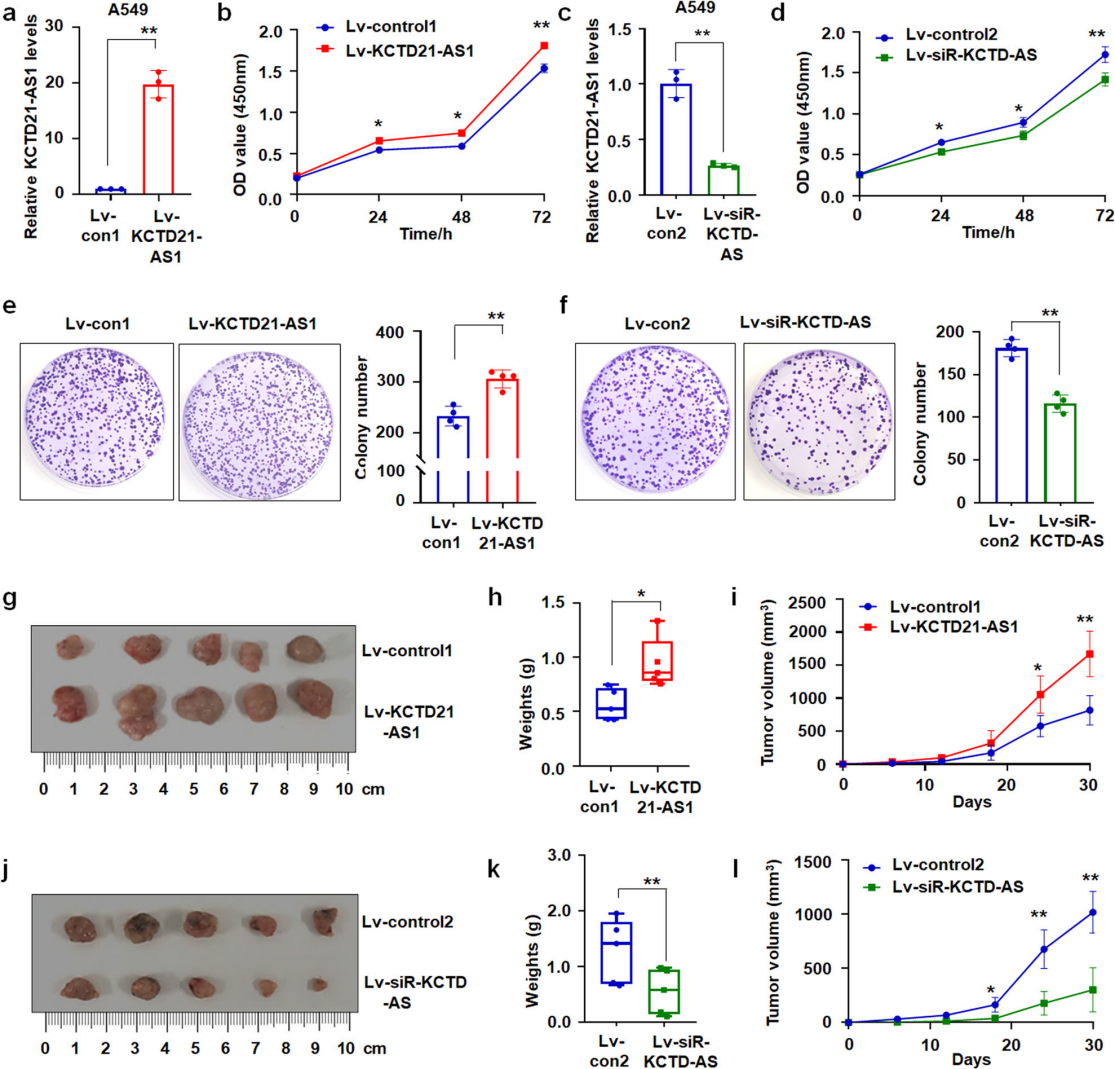
KCTD21-AS1 promoted NSCLC cell proliferation in vitro and in vivo. **a** qRT-PCR results showed KCTD21-AS1 expression in the lv-KCTD21-AS1-treated A549 cells. Data were shown as mean ± SD for triplicate experiments. **p < 0.01; Student’s *t*-test. **b** CCK8 assay illustrated that cell proliferation was promoted in KCTD21-AS1-treated A549 cells. Data were shown as mean ± SD for triplicate experiments. *p < 0.05, **p < 0.01; ANOVA. **c** siRNA inhibited KCTD21-AS1 expression in A549 cells. Data were shown as mean ± SD for triplicate experiments. **p < 0.01; Student’s *t*-test. **d** CCK8 assay. siRNA obviously inhibited A549 cell proliferation. Data were shown as mean ± SD for triplicate experiments. *p < 0.05, **p < 0.01; ANOVA. **e**, **f** The colony numbers of A549 cells infected with lv-KCTD21-AS1 or lv-siR-KCTD21-AS1 were analyzed. Data were shown as the mean ± SD for triplicate experiments. *p < 0.05, **p < 0.01; Student’s *t*-test. **g**–**i** Tumor size, weight, and volume changes of KCTD21-AS1-overexpressed xenografts in nude mice. Data were shown as median (interquartile range) or mean ± SD. n = 5, *p < 0.05, **p < 0.01; Mann–Whitney U test or Student’s *t*-test. **j**–**l** Tumor size, weight, and volume changes of siRNA-treated xenografts were measured. Data were shown as median (interquartile range) or mean ± SD. n = 5, **p < 0.01; Mann–Whitney U test or Student’s *t*-test.

LncRNAs participate in the tumorigenesis and progression of cancers^14^. Next, we detected its expression in cells and constructed lentiviral KCTD21-AS1-overexpressed with siRNA-KCTD-AS vectors as in our previous reports^15,16^ to investigate the roles of KCTD21-AS1 in NSCLC (Supplementary Fig. 1e). The results showed that KCTD21-AS1 expression was higher in NSCLC cell lines than in control BEAS-2B cells (Supplementary Fig. 1f, g). The results of in situ hybridization demonstrated that KCTD21-AS1 was overexpressed in lv-KCTD21-AS1-treated A549 and H1975 cells compared with the control (Fig. 1h; Supplementary Fig. 1h). KCTD21-AS1 overexpression was promoted, but its downregulation inhibited NSCLC cell proliferation compared with the controls (Fig. 2a–d; Supplementary Fig. 2a–d). KCTD21-AS1 overexpression also increased whereas its downregulation decreased the colony number of NSCLC cells (Fig. 2e, f; Supplementary Fig. 2e, f).

Furthermore, the xenografts were produced by subcutaneously injecting A549 cells stably expressing KCTD21-AS1 or siRNA into the backs of BALB/C-nu nude mice to investigate the role of KCTD21-AS1 in vivo. The results showed that the volumes of lv-KCTD21-AS1-treated xenografts considerably increased; the weights evidently increased, and KCTD21-AS1 levels were higher than those in lv-control-treated groups (Fig. 2g, h; Supplementary Fig. 3a). The growth curve demonstrated that KCTD21-AS1 promoted NSCLC cell growth in vivo (Fig. 2i). siRNA-KCTD21-AS1-treated xenografts demonstrated smaller volumes, lighter weights, and slower tumor-growth curve (Fig. 2j–l; Supplementary Fig. 3b). These results indicated that KCTD21-AS1 accelerated NSCLC cell growth.

**Fig. 3:**
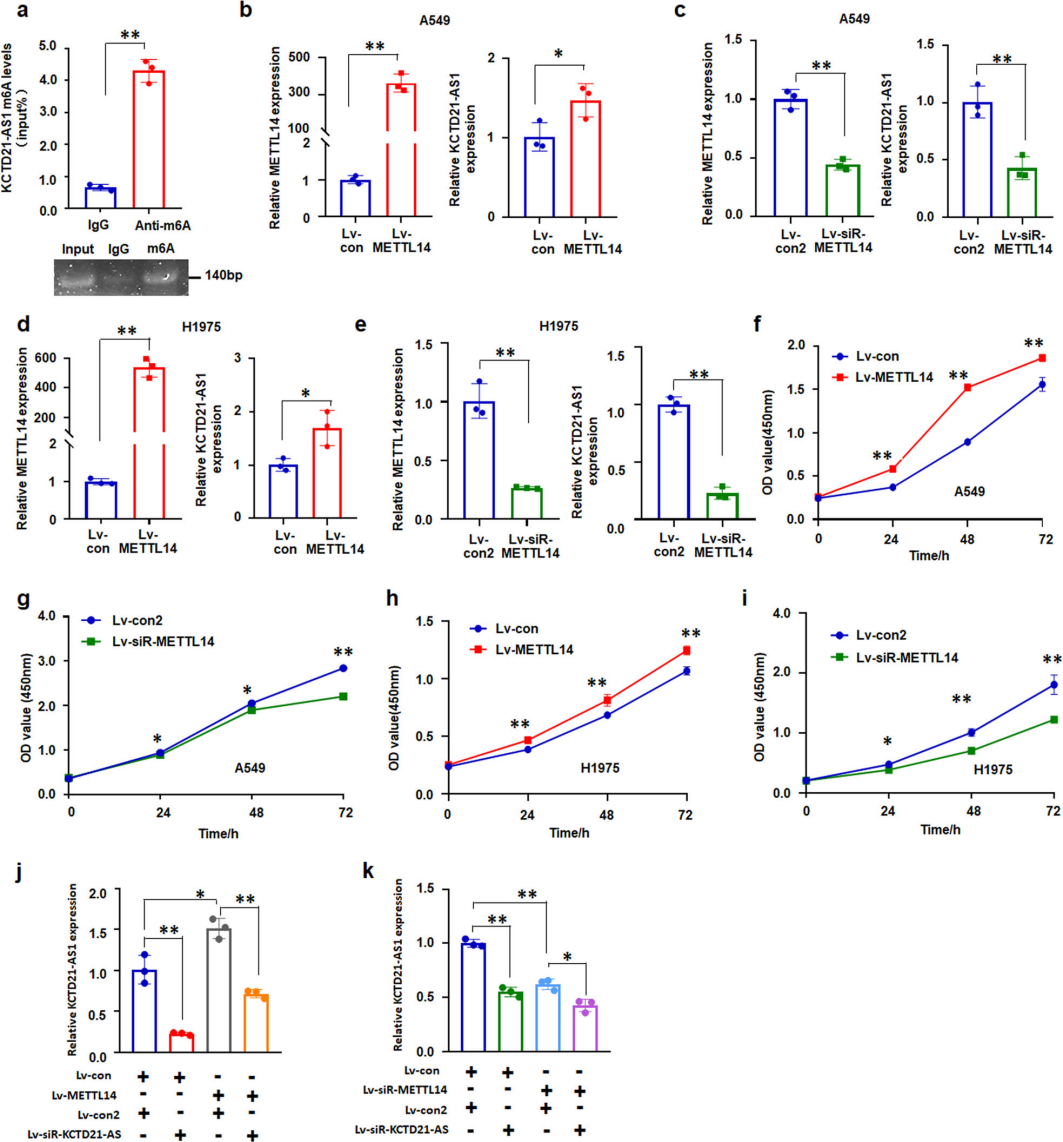
The roles of m6A in regulating KCTD21-AS1 activity. **a** meRIP analysis of m6A-modified KCTD21-AS1 in A549 cells. **b**, **c** METTL14 overexpression increased, but siRNA-METTL14 inhibited KCTD21-AS1 levels in A549 cells. **d**, **e** Effect of METTL14 overexpression and siRNA-METTL14 on KCTD21-AS1 levels in H1975 cells. **f**, **g** CCK8 assay showed METTL14 overexpression promoted, whereas siRNA-METTL14 inhibited A549 cell proliferation. **h**, **i** METTL14 overexpression promoted, whereas siRNA-METTL14 inhibited H1975 cell growth. **j** Blocking KCTD21-AS1 attenuated the roles of METTL14 in increasing KCTD21-AS1 expression. **k** Blocking KCTD21-AS1 accelerated the roles of siRNA-METTL14 in decreasing KCTD21-AS1 expression. Data was presented as mean ± SD for triplicate experiments. *p < 0.05; **p < 0.01; Student’s *t*-test or ANOVA.

m6A methylation, as an epigenetic modification, regulates the function and activity of lncRNAs^17^. METTL14 serves as a core m6A writer to catalyze the methylation of lncRNAs^18^. Then, we investigated the roles of m6A in regulating KCTD21-AS1 activity. The meRIP results showed that anti-m6A pulled down m6A-modified KCTD21-AS1 in A549 cells (Fig. 3a). In NSCLC cells, METTL14 overexpression increased KCTD21-AS1 levels, but siRNA-METTL14 downregulated KCTD21-AS1 levels (Fig. 3b–e). METTL14 overexpression also promoted cell proliferation, but siRNA-METTL14 suppressed the cell proliferation of NSCLC cells (Fig. 3f–i).

Then, siRNA-KCTD21-AS was used to investigate the effects of METTL14 on KCTD21-AS1 expression. The results showed that the promoting role of METTL14 in KCTD21-AS1 expression was suppressed by siRNA-KCTD21-AS treatment (Fig. 3j). Moreover, the suppressive role of siR-METTL4 in KCTD21-AS1 expression was further strengthened by siRNA-KCTD21-AS (Fig. 3k). These results revealed that the m6A modification of KCTD21-AS1 could be affected by METTL14.

### KCTD21-AS1 interacted with miR-519d-5p, which inhibited NSCLC proliferation

**Fig. 4:**
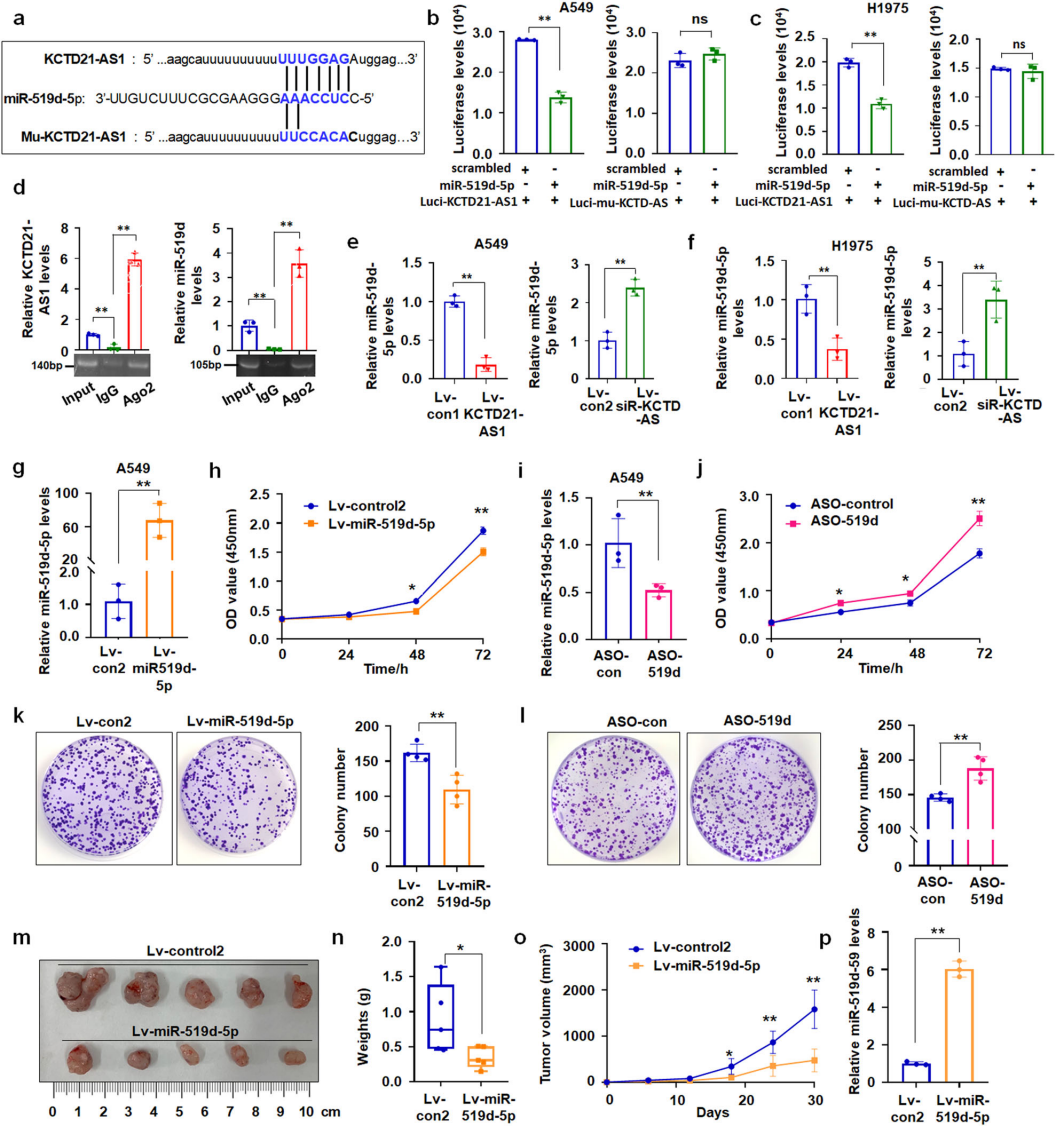
miR-519d-5p suppressed lung cancer cell proliferation. **a** The binding sites between miRNA and KCTD21-AS1. **b**, **c** miR-519d-5p regulated luciferase expression in A549 and H1975 cells. **d** RNA immunoprecipitation assays. miR-519d-5p and KCTD21-AS1 were estimated by qRT-PCR. **e**, **f** KCTD21-AS1 overexpression or siRNA-KCTD21-AS1 regulated miR-519d-5p levels in A549 and H1975 cells, respectively. **g**, **h** miR-519d-5p overexpression inhibited A549 cell proliferation. **i**, **j** miR-519d-5p inhibitor promoted A549 cell proliferation. **k**, **l** miR-519d-5p decreased, whereas ASO-519d-5p increased A549 cell colony formation. **m**–**o** Tumor size, weight, and volume changes were measured in miR-519d-5p- or control-treated xenografts, n = 5. **p** miR-519d-5p levels in xenografts. Data are presented as the mean ± SD or median (interquartile range). *p < 0.05; **p < 0.01; Student’s *t*-test, ANOVA, or Mann–Whitney U test.

LncRNAs are involved in regulating the cell cycle, proliferation, and migration of cancer cells by competing with endogenous RNA (ceRNA)^19^, and lncRNA can act in concert with Ago2 to compete with ceRNA. The binding of miRNAs was analyzed using miRDB bioinformatics prediction, and the results showed that KCTD21-AS1 can potentially bind to miR-519d-5p, miR-922, and miR-5582-3p (Fig. 4a; Supplementary Fig. 4a). Next, luciferase assay demonstrated that the luciferase levels decreased in pc3.1-luci-KCTD21+miR-519d-5p-treated cells, but not in miR-922- or miR-5582-3p- or mutant vector (luci-mu-KCTD-AS, the luciferase expression vector without the binding sites of miRNAs)- treated cells, compared with the controls (Fig. 4b, c; Supplementary Fig. 4b). This finding indicated that KCTD21-AS1 can potentially bind with miR-519d-5p. The levels of KCTD21-AS1 and miR-519d-5p increased the RIPA production pulled down by the anti-Ago2 antibody (Fig. 4d), indicating that KCTD21-AS1 may interact with miR-519d-5p via Ago2. Moreover, miR-519d-5p expression was decreased in lv-KCTD21-AS1-treated cells, but increased in lv-siR-KCTD21-AS1-treated cultures (Fig. 4e, f). miR-519d-5p expression was also reduced in lv- KCTD21-AS1-treated xenografts, but increased in lv-siR-KCTD21-AS1-treated xenografts (Supplementary Fig. 3c, d). These results supported that KCTD21-AS1 regulated miR-519d-5p expression.

Subsequently, we investigated the roles of miR-519d-5p in the proliferation of NSCLC. miR-519d-5p levels decreased in NSCLC cells compared with the BEAS-2B control (Supplementary Fig. 5a). miR-519-5p overexpression significantly inhibited cell growth, whereas the inhibitor ASO-519d enhanced cell growth compared with the controls (Fig. 4g–j; Supplementary Fig. 5b–e). Colony numbers decreased in lv-miR-519-5p-treated cells compared with lv-cons-treated cultures. miR-519-5p downregulation by ASO-519d can increase the colony numbers compared with the controls (Fig. 4k, l; Supplementary Fig. 5f, g). Moreover, A549 cells stably expressing miR-519d-5p were produced to investigate the role of miR-519d-5p in xenografts. The volumes were suppressed; weights decreased; the tumor-growth curve was suppressed, and miR-519d levels were increased in miR-519-5p-treated xenografts compared with the control (Fig. 4m–p).

### KCTD21-AS1 promoting cell proliferation was blocked by miR-519d-5p

The above mentioned results showed that KCTD21-AS1 overexpression can reduce miR-519d-5p levels and promote NSCLC cell proliferation. In studying the regulation of KCTD21-AS1 to miR-519d-5p, we determined whether or not miR-519d-5p recovery can affect the role of KCTD21-AS1 in promoting cell growth. The results demonstrated that miR-519d-5p recovery prevented KCTD21-AS1-promoting cell proliferation compared with lv-con2 (Supplementary Fig. 6a, b). When suppressing miR-519d-5p expression by ASO-519d (inhibitor), the promoting role of KCTD21-AS1 in cell proliferation was further strengthened (Supplementary Fig. 6c, d).

Next, we investigated the role of miR-519d-5p in blocking KCTD21-AS1 action in vivo. Xenografts showed that compared with the control, the volumes were smaller; the weights were lighter; and the tumor-growth curve was slow in lv-KCTD21-AS1+lv-miR-519d-treated xenografts (Supplementary Fig. 6e–g). These results indicated that miR-519d-5p blocked the role of KCTD21-AS1 in promoting cell proliferation.

### KCTD21-AS1 promoted but miR-519d-5p inhibited NSCLC cell metastasis

NSCLC cell migration assay was performed to determine the roles of KCTD21-AS1 and miR-519d-5p in metastasis. The results showed that KCTD21-AS1 overexpression promoted H1975 and A549 cell metastasis, but siRNA-KCTD21-AS1 inhibited cell metastasis (Supplementary Fig. 7a–d). miR-519d-5p treatment suppressed H1975 and A549 cell metastasis, whereas ASO-519d accelerated cell metastasis (Supplementary Fig. 7e–h). Then, the expression of metastasis-related genes (E-cadherin, N-cadherin, vimentin, and α-SMA) was investigated after NSCLC cells were treated with KCTD21-AS1 or miR-519d-5p. We found that KCTD21-AS1 overexpression decreased E-cadherin but increased N-cadherin, vimentin, and α-SMA levels compared with lv-control treatment. siRNA-KCTD21-AS1 treatment increased E-cadherin but decreased N-cadherin, vimentin, and α-SMA levels (Supplementary Fig. 7i). Similar results were also observed in Lv-KCTD21-AS1 overexpression or lv-siR-KCTD21-AS1-treated xenografts (Supplementary Fig. 3g, h). miR-519d-5p treatment increased E-cadherin, but decreased N-cadherin, vimentin, and α-SMA levels. ASO-519d treatment contributed to different results (Supplementary Fig. 7j). Similarly, the results of Lv-519d-5p-treated xenografts supported the regulatory role of miR-519d-5p to these migrative genes (Supplementary Fig. 5i). The results also demonstrated that the recovery of miR-519d-5p inhibited KCTD21-AS1-promoting cell metastasis (Supplementary Fig. 8a, b) and regulated the expression of metastasis-related genes in vitro (Supplementary Fig. 8c) and in vivo (Supplementary Fig. 6k) compared with the control. This finding indicated that miR-519d-5p blocked KCTD21-AS1-promoting cell migration.

**Fig. 5:**
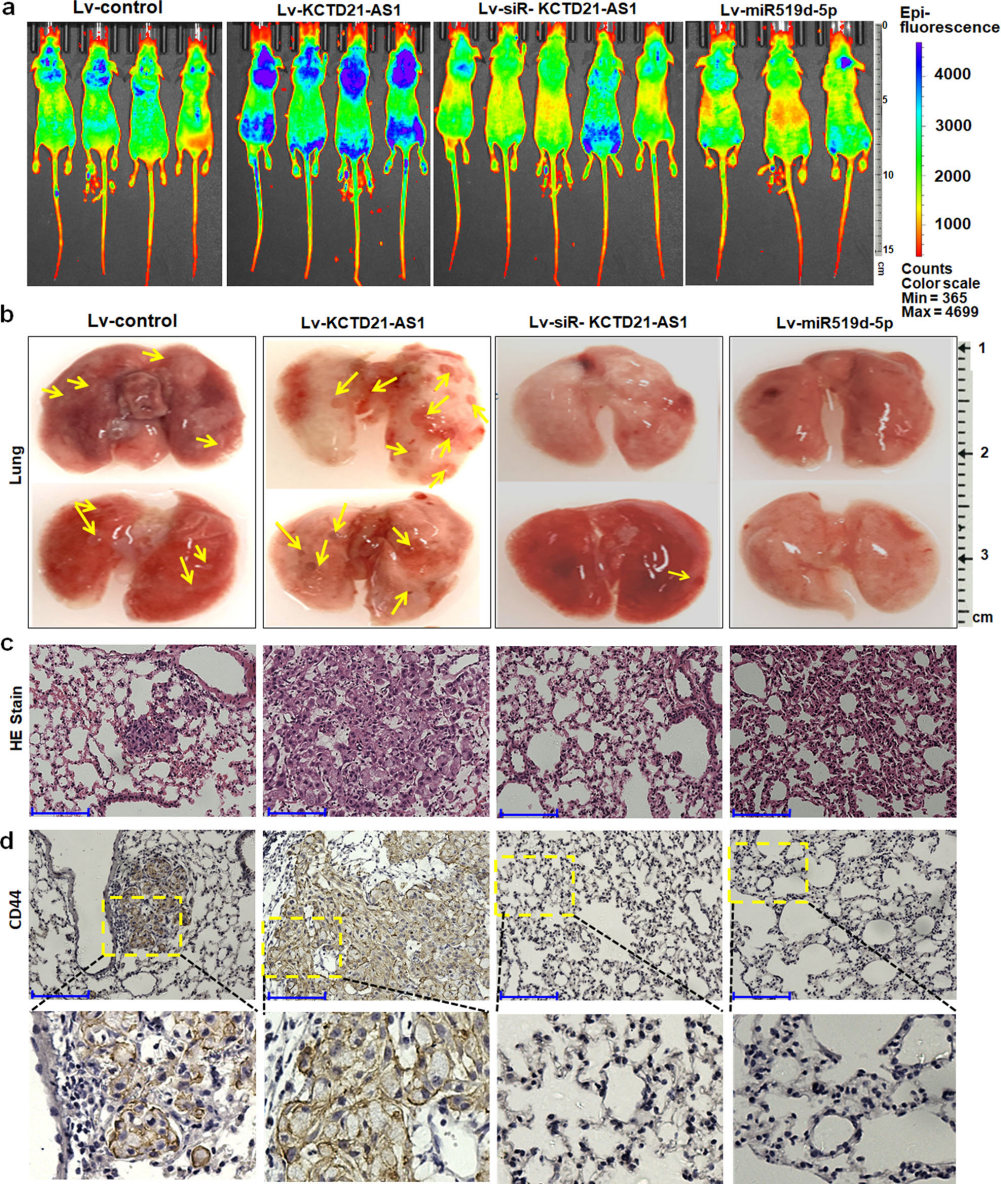
The role of miR-519d-5p and KCTD21-AS1 in tumor metastasis. **a** The experimental metastasis mouse model was injected with KCTD21-AS1, siRNA-KCTD21-AS1, miR-519d-5p-treated A549/GFP cells. **b** Visualization of the metastatic tumors to lungs. **c** Visualization of the HE-stained lung section. Bar = 125 μm. **d** Immunohistochemistry of human CD44 staining. Bar = 125 μm.

Furthermore, we investigated the roles of KCTD21-AS1 and miR-519d-5p in regulating NSCLC cell metastasis in vivo. The results showed that more NSCLC cells migrated into the lung tissues of lv-KCTD21-AS1-treated groups compared with the control, whereas siRNA-KCTD21-AS1 and miR-519d-5p treatment inhibited the migration of NSCLC cells into the lung tissue (Fig. 5a, b; Supplementary Fig. 9a). HE staining showed that more lung cancer cells grew in the lung tissues of lv-KCTD21-AS1-treated groups, and siRNA-KCTD21-AS1 and miR-519d-5p suppressed cancer cell proliferation in the lung tissues compared with the control treatment (Fig. 5c; Supplementary Fig. 9b). Human CD44 straining was further used to prove that the human lung cancer cells migrated into lung tissues of mice. The results showed that more CD44-positive human lung cancer cells grew in lv-KCTD21-AS1-treated groups, but relatively fewer CD44-positive cancer cells were revealed in the siRNA-KCTD21-AS1- or miR-519d-5p-treated groups compared with the controls (Fig. 5d). These results indicated that KCTD21-AS1 can promote NSCLC cell metastasis, whereas miR-519d-5p can suppress such a process.

### miR-519d-5p affected lung cancer cell autophagy through TIPRL

**Fig. 6:**
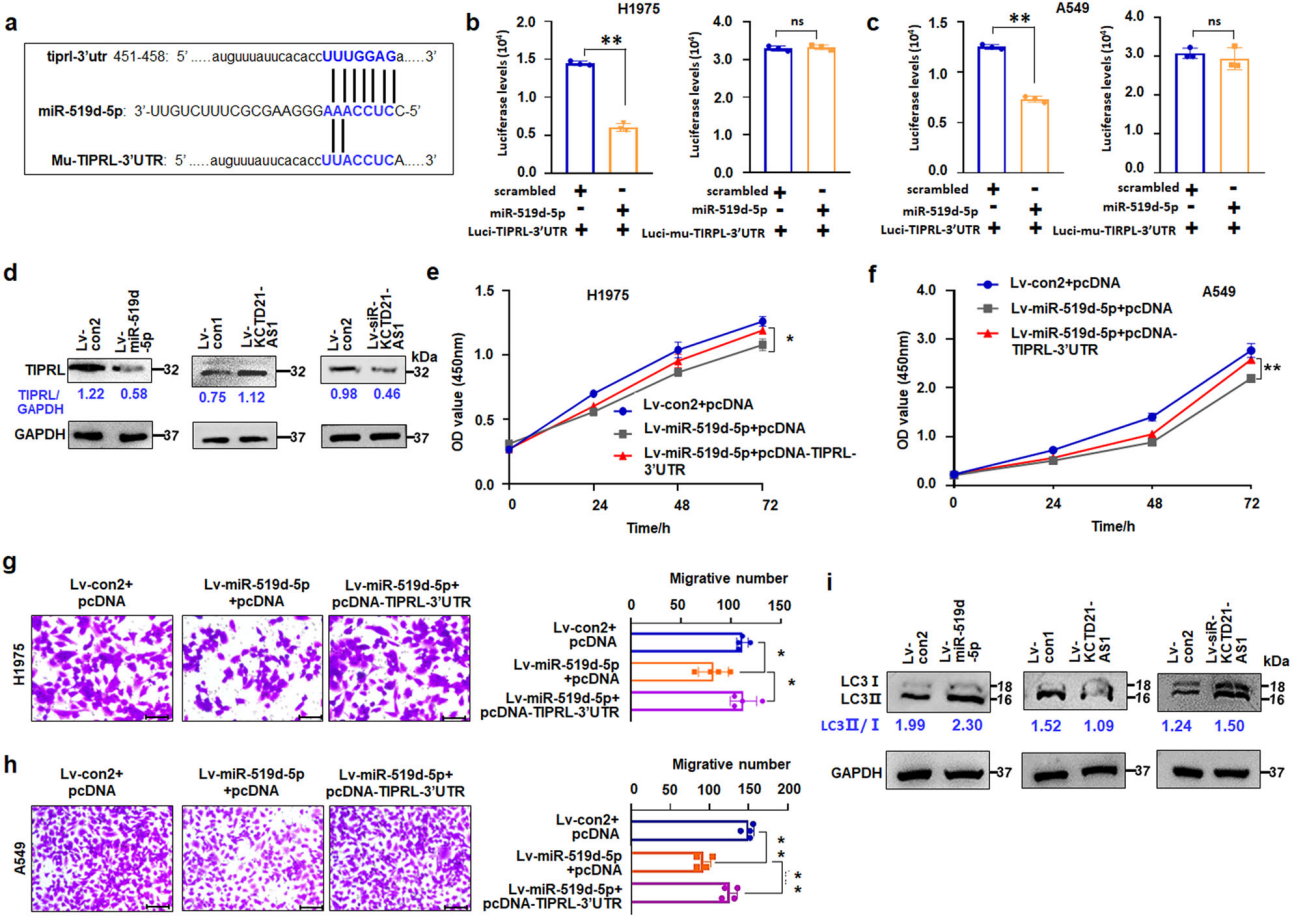
KCTD21-AS1 and miR-519d-5p regulated TIPRL expression. **a** The sites of TIPRL-3’-UTR or Mu-TIPRL-3’-UTR targeted by miR-519d-5p. **b**, **c** Luciferase levels were analyzed in both pc3.1-luci-TIPRL-3’-UTR and miR-519d-5p-treated H1975 or A549 cells, respectively. Data were expressed as mean ± SD for triplicate experiments. **p < 0.01, Student’s *t*-test. **d** immunoblotting detection from different membrane, and the sample loading amount according to the reference control, which was run on a different gel than the corresponding sample of interest. **e**, **f** Effect of TIPRL recovery on the role of miR-519d-5p in H1975 or A549 cell growth using CCK8 assay, respectively. Data were expressed as mean ± SD for triplicate experiments. *p < 0.05, **p < 0.01; ANOVA. **g**, **h** Effect of TIPRL-3′UTR recovery on miR-519d-5p-regulating H1975 or A549 cell metastasis, respectively. Data were expressed as mean ± SD for triplicate experiments. Bar = 100 μm. *p < 0.05, **p < 0.01; ANOVA. **i** immunoblotting detection from different membrane, and the sample loading amount according to the reference control, which was run on a different gel than the corresponding sample of interest.

lncRNAs can modulate gene expression through interaction with miRNA^20^. The above mentioned results revealed that KCTD21-AS1 can regulate miR-519d-5p. Then, we investigated the regulatory genes of KCTD21-AS1 and miR-519d-5p. First, the results revealed that miR-519d-5p potentially bound the gene sequences of the TOR signaling pathway regulator (TIPRL)−3ʹ-UTR by TargetScanHuman analysis (http://www.targetscan.org). Next, the regulation of miR-519d-5p to TIPRL was investigated, and luciferase assay showed that miR-519d-5p decreased the luciferase levels in Luci-TIPRL-3’UTR- and miR-519d-5p- transfected cells. However, it did not reduce the expression of luciferase in Luci-Mu-TIPRL-3’-UTR (mutant 3’-UTR vector)-treated cultures (Fig. 6a–c). The immunoblotting results showed that TIPRL protein expression was reduced in miR-519d-5p-treated cultures (Fig. 6d) and xenografts (Supplementary Fig. 5h, i), indicating that miR-519d-5p can negatively regulate TIPRL expression through binding TIPRL-3ʹ-UTR.

Considering that miR-519-5p can suppress NSCLC growth, we then investigated the effect of TIPRL-3ʹ-UTR recovery on the role of miR-519d-5p in regulating cell growth and migration. The results demonstrated that TIPRL-3ʹ-UTR treatment attenuated miR-519d-5p-suppressing cell proliferation compared with the control (Fig. 6e, f). Similar results were found in the migration study (Fig. 6g, h). These results supported that miR-519d-5p can regulate cell growth and migration by negatively regulating TIPRL expression.

We also investigated the effect of KCTD21-AS1 on regulating TIPRL expression. Immunoblotting results showed that KCTD21-AS1 overexpression promoted, but its downregulation suppressed TIPRL expression in cells (Fig. 6d) and xenografts (Supplementary Fig. 3g, h). miR-519d-5p treatment attenuated the role of KCTD21-AS1 in regulating TIPRL expression (Supplementary Fig. 6k). These results indicated that KCTD21-AS1 can affect TIPRL expression though miR-519d-5p.

TIPRL, an essential PP2A inhibitory protein, plays an important role in cell apoptosis and cell proliferation. The protein is upregulated in various carcinomas, including NSCLC^21^. TIPRL affects cell growth and autophagy through light chain 3 (LC3)-II/I^22^. Then, the results demonstrated that LC3-II/I was found to be increased in miR-519d-5p-treated cells compared with the control. KCTD21-AS1 overexpression downregulated the LC3-II/I ratio, whereas siRNA-KCTD21-AS1 increased the LC3-II/I ratio (Fig. 6i). These results supported that miR-519d-5p may promote cell autophagy through regulating TIPRL, whereas KCTD21-AS1 may inhibit such a process.

### miR-519d-5p promoted the phagocytic function of macrophages through CD47

**Fig. 7:**
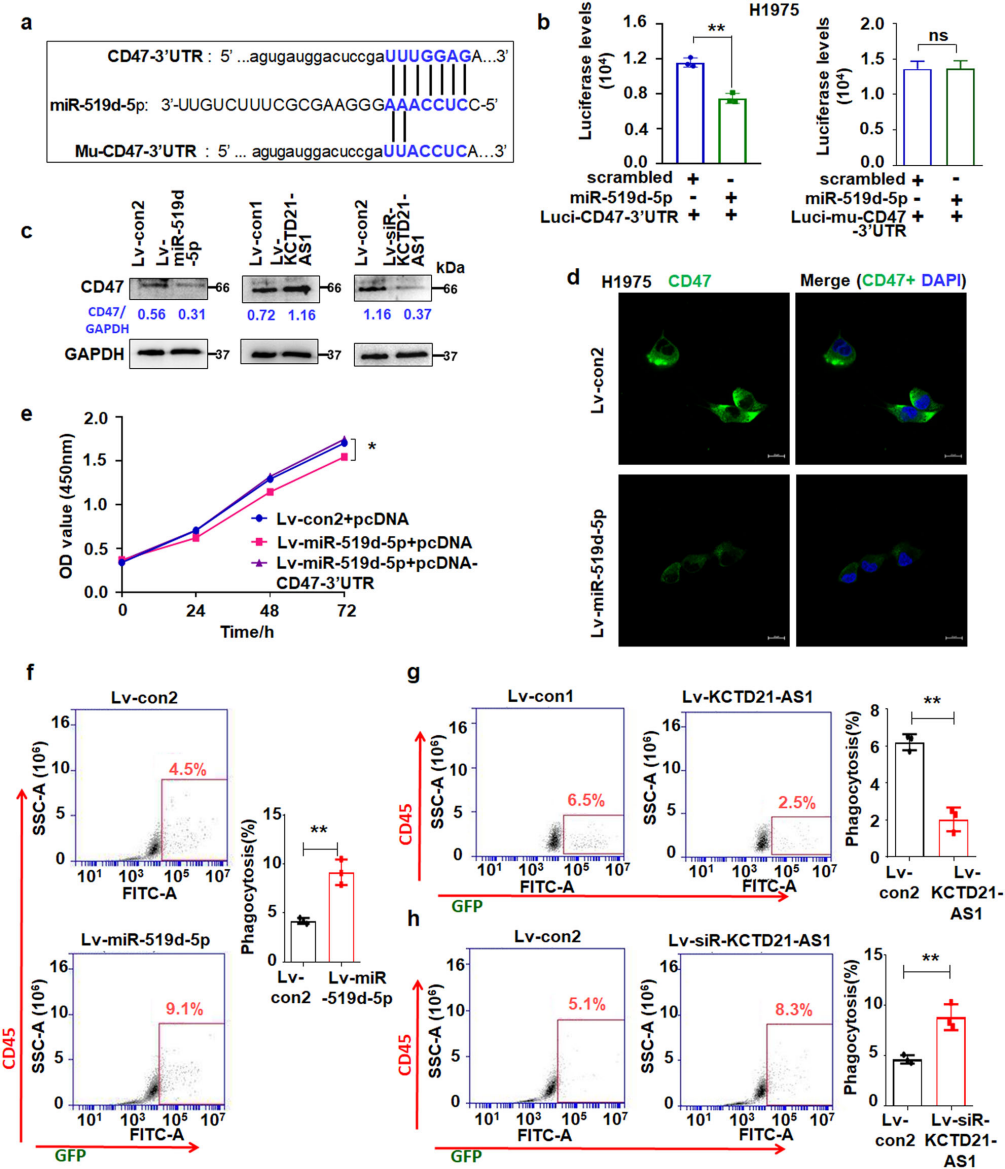
KCTD21-AS1 and miR-519d-5p regulated CD47 expression. **a** CD47-3’-UTR or Mu-CD47-3’-UTR targeted by miR-519d-5p. **b** Luciferase levels were analyzed in both pc3.1-luci-CD47-3’-UTR and miR-519d-5p-treated H1975 cells. Data were expressed as mean ± SD for triplicate experiments. **p < 0.01, Student’s *t*-test. **c** immunoblotting detection from different membrane, and the sample loading amount according to the reference control, which was run on a different gel than the corresponding sample of interest **d** immunofluorescence analysis of CD47 levels in H1975 cells. Bar = 20 μm. **e** Effect of CD47-3′UTR recovery on the role of miR-519d-5p in H1975 cell growth using CCK8 assay. Data were expressed as mean ± SD for triplicate experiments. *p < 0.05; ANOVA. **f** lv-miR-519d-5p treatment enhanced the phagocytic function of macrophages. Data were expressed as mean ± SD for triplicate experiments. **p < 0.01, Student’s *t*-test. **g** KCTD21-AS1 overexpression reduced the phagocytic function of macrophages. **h** siRNA-KCTD21-AS1 promoted the phagocytic function. Data were expressed as mean ± SD for triplicate experiments. **p < 0.01, Student’s *t*-test. Data were expressed as mean ± SD for triplicate experiments. **p < 0.01, Student’s *t*-test.

The TargetScanHuman analysis results also revealed that miR-519d-5p potentially bound the 3ʹ-UTR sequence of CD47 (Fig. 7a). Luciferase expression showed that miR-519d-5p decreased the luciferase levels in Luci-CD47-3’-UTR- and miR-519d-5p- treated cells, but the Luci-Mu-CD47-3’-UTR (mutant 3’-UTR vector) treatment did not change the luciferase levels compared with the controls (Fig. 7b). Immunoblotting and fluorescence analysis showed that CD47 expression was reduced in miR-519d-5p-treated NSCLC cells compared with the controls. KCTD21-AS1 overexpression increased, whereas its downregulation inhibited CD47 levels in cells (Fig. 7c, d). CD47-3ʹ-UTR recovery attenuated miR-519d-5p-suppressing cell proliferation and migration compared with the control (Fig. 7e, Supplementary Fig. 10a), indicating that miR-519d-5p can affect cell growth and migration by regulating CD47 expression.

Innate immune checkpoints, such as the CD47/SIRPα axis, PD-1/PD-L1 axis, and MHC-I/LILRB1 axis, play important roles in tumor-mediated immune escape from the clearance by phagocytosis^23^. Then, human peripheral blood mononuclear cells (PBMC) were sorted, and macrophages were induced (Supplementary Fig. 10b) and co-cultured with A549/GFP+ cells. The results showed that lv-miR-519d-5p treatment enhanced the phagocytic function compared with the control (Fig. 7f). KCTD21-AS1 overexpression reduced the phagocytic function of macrophages, whereas its downregulation promoted such a function (Fig. 7g, h).

### miR-519d-5p, TIPRL, and CD47 levels in NSCLC tissues

**Fig. 8:**
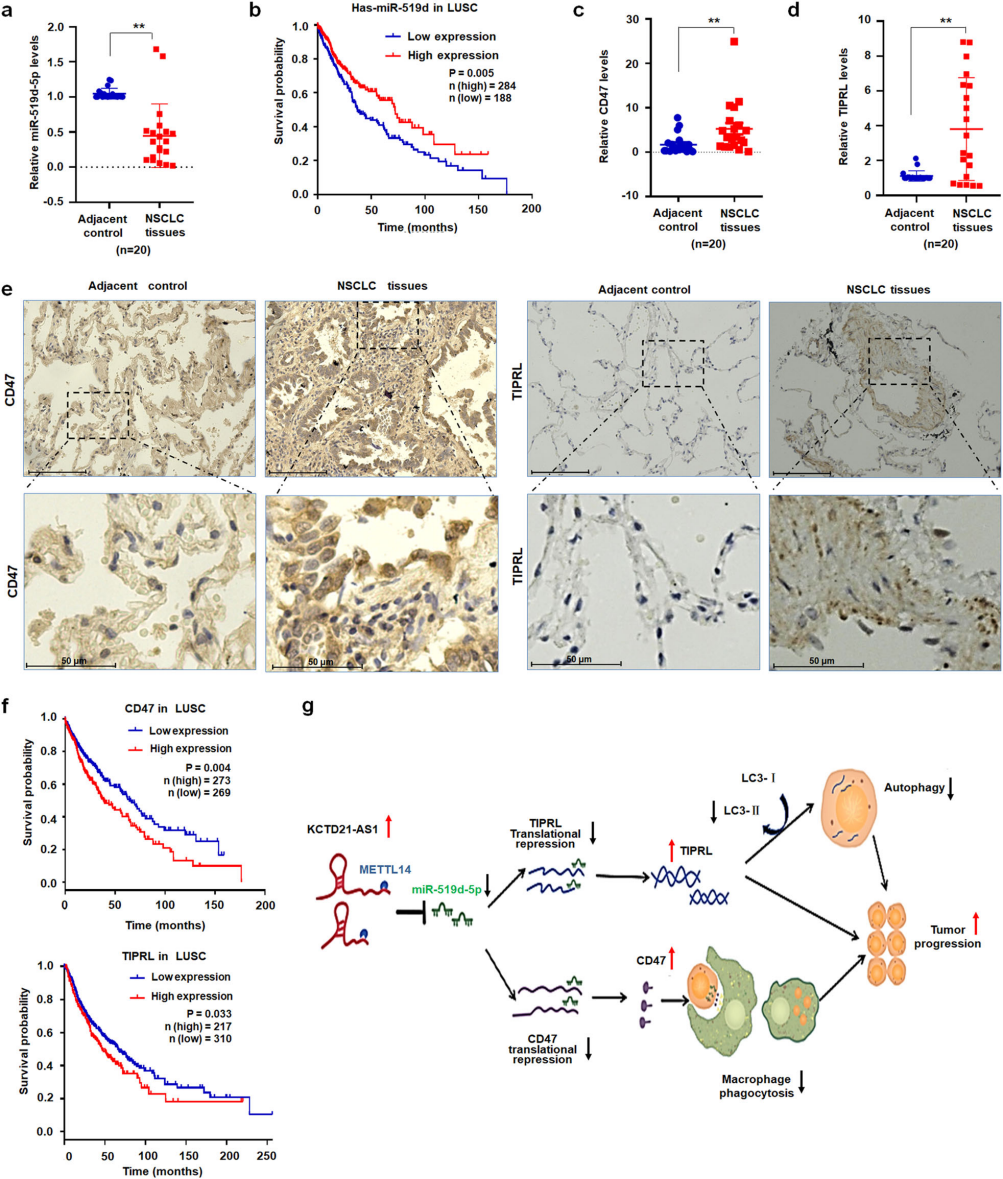
Levels of miR-519d-5p and its targets in NSCLC. **a** qRT-PCR results of miR-519d-5p in NSCLC tissues (n = 20). **b** Kaplan–Meier plot analysis of miR-519d-5p levels with the overall survival of patients with squamous carcinoma (LUSC). **c**, **d** qRT-PCR analysis of CD47 and TIPRL in NSCLC tissues (n = 20). **e** Immunohistochemistry showed cd47 and TIPRL levels are high in NSCLC tissues versus adjacent control, bar = 125 μm (upper); bar = 50 μm (below). **f** Kaplan–Meier plotter analysis of CD47 and TIPRL expression with the overall survival of patients with NSCLC (LUSC). **g** Proposed model by which miR-519d-5p, regulated by KCTD21-AS1, promoted the phagocytic function of macrophages and suppressed lung cancer cell proliferation. Data were shown as median (interquartile range), **p < 0.01, Mann–Whitney U test.

The above-mentioned results indicated that miR-519d-5p can effectively suppressed NSCLC cell proliferation and metastasis. However, the role of miR-519d-5p in the pathogenesis of NSCLC requires clarification. First, the miR-519d-5p levels in carcinoma tissue lysates prepared from 20 patients with NSCLC were further analyzed. Compared with lung adjacent control samples, miR-519d-5p levels were reduced in lung carcinoma samples (Fig. 8a). The Kaplan–Meier plot showed that low miR-519d-5p levels in patients with squamous carcinoma corresponded with poorer survival than high levels (http://www.kmplot.com/, Fig. 8b). As the targets of miR-519d-5p, the levels of TIPRL and CD47 of NSCLC tissues were relatively higher in NSCLC tissues than in adjacent control samples through qRT-PCR and IHC analyses, respectively (Fig. 8c–e). Second, the Kaplan–Meier plot further showed that high TIPRL (http://www.kmplot.com/) or CD47 (gene expression RNAseq from GDC TCGA LUSA, https://xenabrowser.net/) levels in patients with NSCLC corresponded with poorer survival than low levels (Fig. 8f). These results supported that miR-519d-5p played tumor suppressive roles, but CD47 and TIPRL promoted tumorigenesis in NSCLC.

## Discussion

Cancer cells can evade immune surveillance via the immune checkpoint CD47/SIRPα, and blocking this immune checkpoint is a useful strategy to engineer macrophages for cancer immunotherapy^24^. Given the limited understanding of the regulation of CD47 and the function of TAMs, the immunosuppressive status of CD47-related factors remains poorly understood. In this study, we revealed that KCTD21-AS1 inhibited the phagocytic function of macrophages through CD47, whereas miR-519d-5p promoted such a function. In addition, KCTD21-AS1 promoted lung cancer proliferation by regulating TIPRL, whereas miR-519d-5p suppressed this proliferation (Fig. 8g).

The immune cells within TME play crucial roles in the tumorigenesis of different kinds of tumors. TME contains a series of immune cells, cancer-associated fibroblasts, and endothelial cells, and a crosstalk was observed between cancer cells and immune cells^25^. In contrast to traditional radiotherapy or chemotherapy, immunotherapy primarily exert its effect through the culture and modification of the immune cells to further recognize and attack tumor cells with higher specificity and lower side effect^26^. Based on tumor immune evasion, a series of immunotherapy approaches such as blocking CD47/SIRPα has been developed and clinically applied to tumor therapy^27^.

Tumor cells express “self” signals (e.g., CD47 molecules) and prevent the phagocytosis of macrophage. The siRNA specific to CD47 increases the phagocytosis of tumor cells by macrophages^28^. Noncoding RNAs have also been used to regulate CD47 expression to further promote the phagocytotic function of macrophages. Tan et al. demonstrated that miR-708 directly binds to CD47 and plays an important tumor suppressive role in the self-renewal of breast cancer stem cells and TAM-mediated phagocytosis^29^. Similarly, by directly targeting and regulating CD47, miR-708 may serve as an effective and attractive candidate for the immunotherapy of T-ALL^30^. miR-340 overexpression can downregulate *CD47* and inhibit tumor growth via regulating macrophage phagocytosis. MiR-340 also promotes the function of macrophages in tumor immune microenvironments and increases CD8^+^ T cells^31^. In the present study, we demonstrated that miR-519d-5p potentially bound the 3ʹ-UTR sequence of CD47 to further downregulate CD47 expression. Through ceRNA with miR-519d-5p, KCTD21-AS1 treatment suppressed the numbers of phagocytosis of macrophages compared with the control, indicating that KCTD21-AS1 promoted the tumorigenesis of lung cancer via miR-519d-5p-blocking CD47.

LncRNAs play an important role in human cancer types via ceRNAs to regulate targeted gene expression^32^. lncRNA MIAT can reportedly suppress EZH2 expression and promote papillary thyroid carcinoma cell invasion via miR-150^33^. The downregulation of lncRNA NEAT1 increases miR-381 and reduces IGF1 levels, thereby suppressing the apoptosis of ovarian granulosa cells^34^. Here, in situ hybridization results showed that cytosolic presence of KCTD21-AS1 was higher in NSCLC tissues compared with adjacent control, indicating that KCTD21-AS1 might regulate gene expression by ceRNA. In vitro results also demonstrated that the cytosolic and nuclear KCTD21-AS1 levels were higher in KCTD21-AS1-overexpressed cells compared with control treatment. Higher levels of KCTD21-AS1 in cytoplasm in vitro may be related to regulate gene expression, and the higher levels of KCTD21-AS1 in nucleus in vitro may be attributed to high transcription levels of KCTD21-AS1 lentiviral vector. Our results further demonstrated that KCTD21-AS1 regulated TIPRL expression and promoted lung cancer proliferation via ceRNA with miR-519d-5p.

TIPRL, an essential PP2A inhibitory protein, increases in various cancers, including NSCLC and hepatocellular carcinomas^21^. TIPRL plays important roles in cell apoptosis and proliferation of cancer through the TIPRL/PP2A axis. The depletion of circ_0010235 or gain of miR-433-3p would repress NSCLC cell proliferation and autophagy by regulating TIPRL^22^. TIPRL upregulation potentially brings metabolic benefits to lung cancer cells and promotes cancer cells survival^35^. TIPRL downregulation significantly reduces LC3 and CD133 expression, indicating that the TIPRL/LC3/CD133 complex may serve as a valuable biomarker for liver cancer diagnosis^36^. Our results demonstrated that TIPRL was high in NSCLC tissues, which correlated with the poor overall survival of patients with lung cancer. The downregulation of TIPRL evidently increased the LC3-II levels in miR-519d-5p-treated NSCLC cells, indicating that miR-519d-5p suppressing NSCLC proliferation may be related to cell autophagy.

This study demonstrated that KCTD21-AS1 promoted NSCLC cell proliferation and metastasis by regulating CD47 levels, whereas miR-519d-5p inhibited such a processes. Moreover, the oncogenic role of KCTD21-AS1 was related to TIPRL expression regulation via miR-519d-5p, thereby affecting cell autophagy. Our results provided a strategy through miR-519-5p gain or KCTD21-AS1 depletion for NSCLC therapy by regulating CD47 and TIPRL.

## Methods

### Clinical specimens

A total of 20 specimens from patients with NSCLC after operation were collected from Yantai Mountain Hospital (Supplementary Table 1). The collected clinical tumors and their adjacent tissues were stored at −80 °C. This study was approved by the Binzhou Medical College Ethics Committee. Prior to inclusion in the study, patients were fully informed of the study procedure, and they signed a written informed consent. All ethical regulations relevant to human research participants were followed.

### RNA extraction and qRT-PCR

Total RNA (or small RNA) from tissues and cells was extracted by RNAiso Plus (Takara, Dalian, China), and <1 μg of the extracted RNA was reverse transcribed into cDNA by using the SPARKscript II RT Plus Kit (AG0304-B, SpakeJade, Jinan, China). Quantitative real-time PCR was performed using the Quantitect SYBRGreen Kit (Takara) and a StepOnePlus Real-time system (Thermo Fisher, Shanghai, China). GAPDH and 5S gene served as an endogenous control gene. The primers used to amplify miR-519d-5p, TIPRL, KCTD21-AS1, etc. are described in Supplementary Table 2.

### Cell culture and lentiviral vectors

The human cell lines, including NSCLC cells (A549 and NCI-H1975), normal lung epithelial cells (BEAS-2B as NSCLC cell control) and 293T, were obtained from Shanghai Institute of Cell Biology, China. Cells were cultured in RPMI-1640 medium (Gibco, USA) or DMEM medium, (high glucose; Hyclone, USA) supplemented with 10% fetal bovine serum (Gibco). All cells were cultured in an atmosphere containing 5% CO_2_ at 37 °C.

Lentiviral-mediated KCTD21-AS1-overexpressed and siRNA-KCTD-AS vectors were constructed and produced according to the previous report^15^. B*amH* I–E*coR* I element containing KCTD21-AS1 was inserted into FUGW vector to construct the overexpression vector. U6 promoter was used to drive siRNA expression, which was cloned into FUGW vector by X*ba* I and X*ho* I. 293T cells were cultured to package lentivirus. In brief, viral vector (1 mg), the gag/pol expression vector (△8.9, 0.9 mg), and VSVG vector (0.1 mg) were transfected 293 T cells. The virus supernatant was then harvested.

### Cell transfection

Small interfering RNA, miR-519d-5p, miR-519d inhibitor (ASO-519d) and control mimic were synthesized by Shanghai GenePharma. A549 or NCI-H1975 cells were plated at 16 h before transfection, and cells were transfected using Lipofectamine TM 2000 (Invitrogen).

### CCK-8 assay

After transfection, about 1 × 10^3^ A549 and H1975 cells were seeded in each well of 96-well plates. A CCK-8 assay reagent (10 μL) was added into each well after the cells were attached to the well. After 2 h, the culture plate was removed, and the OD value was measured at 450 nm. Each experiment was repeated at least three times.

For gene recovery experiment, TIPRL-3ʹ-UTR or CD47-3’-UTR were cloned by PCR (the primers were shown in Supplementary Table 2). The UTRs (TIPRL-3ʹ-UTR,1300 bp and CD47-3ʹ-UTR, 388 bp) were inserted into pcDNA3.1(+) vector to form the pcDNA-TIPRL-3ʹ-UTR (or CD47-3’-UTR) vector by E*coR* I and X*ho* I. Then, the cells were treated with lv-miR-519d+pcDNA-TIPRL-3ʹ-UTR (or CD47-3’-UTR) to investigate the effect of TIPRL-3ʹ-UTR (or CD47-3’-UTR) on miR-519d-5p regulating cell proliferation using CCK-8 assay.

### Cell transwell assay

A549 and H1975 cells were collected and re-suspended in a serum-free culture medium. The suspension was adjusted to 1 × 10^5^ cells/mL, and 100 μL of cell suspension was directly added into the upper chamber of the transwell culture plate (Corning, NY, USA). The lower chamber contains 600 μL of 30% FBS medium. The subsequent processing is according to the previous reports^37,38^.

### Colony formation

A549 and H1975 cells were collected after transfection. About 1 × 10^3^ cells were added to a 10 cm dish and cultured continuously for 12–14 days. The colonies were washed with PBS and stained with 1% crystal violet, and the colony formation of cells was observed and counted as previously described in refs. ^37,39^.

### In situ hybridization

The 5′-Cy3 labeled probe of KCTD21-AS1 was designed and synthesized by GenePharma (Shanghai, China). In accordance with the instructions of the FISH kit (no. F11201, GenePharma, Shanghai, China), cells were fixed with 4% paraformaldehyde for 20 min. After membrane-disruption treatment, cells were incubated with 1 μM probe and hybridized at 37 °C overnight. Nuclei were stained with DAPI for 2–3 min. Cells were observed under a confocal microscope (Leica TCS SPE, Leica, Dresden, Germany). The tissue sections were dewaxed before incubation, treated with proteinase K, denatured, and incubated with 1 μM probe before hybridizing at 37 °C overnight. Nuclei were stained with DAPI.

### Hematoxylin–eosin (HE) staining and immunohistochemistry (IHC) analysis

Xenografts or tissues were fixed with 4% paraformaldehyde and embedded with paraffin. Then, the sections were dewaxed in xylene and rehydrated in alcohol. Antigen retrieval was performed using sodium citrate, and then endogenous peroxidase was blocked. The sections were incubated with specific rabbit anti-human CD44 (1:100, Boster Biological Technology, China), and TIPRL (1:200, Abcam, USA) at 4 °C overnight. After incubation with secondary antibodies at 37 °C for 1 h, DAB was used for detection. The sections were observed under an EVOSTM M7000 Imaging System (Thermo Fisher Scientific, USA).

### Immunofluorescence staining

Cells were fixed with 4% paraformaldehyde in PBS, permeabilized with 0.5% Triton X-100, and incubated with rabbit anti-human CD47 (1:200 dilution; Boster Biological Technology, China) at 4 °C overnight. Then, cells were incubated with Alexa Fluor 488 donkey anti-rabbit IgG (H + L) at 37 °C for 1 h. Immunofluorescence images were captured under a microscope (DM6000B, Leica) in accordance with a previous study^16^.

### Immunoblotting

Immunoblotting was performed as previously described in refs. ^16,37^. Cell proteins were collected using 1×RIPA buffer (P0013B, Beyotime, Shanghai, China). Then, the protein was isolated by 10% SDS-PAGE and transferred onto a polyvinylidene fluoride membrane, which was blocked with 5% milk and incubated with primary and secondary antibodies. The enhanced chemiluminescence substrates (P0018M, BeyoECL Plus, Beyotime, China) were used to analyze the proteins. The antibodies were as follows: mouse anti-human E-cadherin (1:500, 20874-1-AP, Proteintech, USA), rabbit anti-human N-cadherin (1:500, 22018-1-AP, Proteintech), rabbit anti-human TIPRL (1:5000, AB70795, Abcam, MO, USA), rabbit anti-human α-SMA (1:500, BS70000, Biowworld, MN, USA), rabbit anti-human vimentin (1:500,BS1855, Biowworld), rabbit anti-human METTL14 (1:500, 31591, SAB, Nanjing, China), rabbit anti-human CD47 (1:500, A00360-2, Boster), rabbit anti-human LC3B (1:500, BM4827, Boster), rabbit anti-human GAPDH (1:3000, AP0063), and mouse anti-human GAPDH (1:3000, MB001, both from Biowworld Technology).

### RNA immunoprecipitation (RIP) and N6-methyladenosine (m6A) methylated RIP (MeRIP)

RIP and MeRIP assays were performed using a Magna RIP RNA-binding protein immunoprecipitation kit (17-700, Millipore, Billerica, USA) according to the manufacturer’s instructions^38,40^. The AGO2 antibody used in the experiment was from CST (2897, Boston, MA, USA), and the m6A antibody was from CST (56593, Boston, MA, USA). The co-precipitated RNA was used for cDNA synthesis and evaluated by qRT-PCR.

### Luciferase assay

Luciferase levels were analyzed using a luciferase assay system (E1500, Promega, WI, USA). The wild-type, mutant fragments of KCTD21-AS1 or mRNA 3′-UTR fragments were cloned into pcDNA3.1-luci vector. Cells were transfected with the pcDNA3.1-luci vector or control and lysed at room temperature for 15 min, and the supernatants were collected. Next, 70 μL of lysate were added onto a 96-well plate, and then 20 μL of luciferase assay reagent II was added. The luciferase activity was determined using a chemiluminescence analyzer (Infinite 200 PRE, Tecan Austria GmbH).

### Migratory cancer cell detection in vivo

A549-GFP cells were infected with lentivirus, and 1.5 × 10^6^ cells were collected. Then, the cells were injected into the tail vein of nude mice. After 6–7 weeks, the migration of GFP positive cells was observed.

### Macrophage extraction

This study was approved by the Binzhou Medical College Ethics Committee. Human peripheral blood was collected from a central blood station in Yantai. Prior to inclusion in the study, volunteers were fully informed of the procedure, and they signed a written informed consent. Human PBMCs were obtained after being treated with lymphocyte (P8900, Solarbio China). In accordance with the instructions, CD14^+^CD16^-^ monocytes were obtained by sorting using the EasySep Human Monocyte Isolation Kit (19359, STEMCELL, USA). Afterward, the sorting efficiency of monocytes was measured by flow cytometry.

### Phagocytic function of macrophages

Macrophages were collected at the 5th day after stimulation of differentiation, and A549-EGFP cells were collected. Then, 5 × 10^4^ macrophages and 10×10^4^ A549-EGFP cells were inoculated onto a 48-well plate. After co-culture for 1 h, the suspension cells were washed, the adherent cells were collected, and APC anti-Human CD45 antibody was added. The cells were washed with PBS and resuspended. For the detection of phagocytosis, 10,000 cells per sample were analyzed using a FACS flow cytometer (BD, USA), and unstained control and single stained cells were prepared for gating. After CD45^+^ macrophages were selected using the APC fluorescent channel gate, and the phagocytosis was detected by selecting CD45^+^GFP^+^cells using the FITC channel. Therefore, phagocytosis was calculated as the percentage of APC^+^GFP^+^ cells among APC^+^ macrophages^31^.

### Xenograft mouse model

A549 cells were treated with lentivirus and stably expressed KCTD21-AS1 or miR-519d-5p. Then, 1 × 10^7^ cells were injected subcutaneously into the shoulder and back of BALB/c nude mice (female, 6–8 weeks old; HFK Bio-Technology, Beijing, China). The tumor width and height were measured using a vernier scale, and tumor volume was calculated using the following formula: tumor volume = (length × width^2^)/2. About a month later, the mice were euthanized with intraperitoneal injection of barbiturate. All animal experiments were approved by the Ethics Committee of Binzhou Medical University. We have complied with all relevant ethical regulations for animal use.

### Statistics and reproducibility

SPSS 22.0 (IBM Corp., Armonk, NY, USA) was used to analyze experimental data. Normally distributed data are shown as the mean ± SD. Two averages and multiple groups were analyzed by Student’s *t* test and ANOVA, respectively. For homogeneous variance assumption, the LSD test or Games–Howell test was used to compare the means. Abnormally distributed data are shown as median (interquartile range), and analyzed by Mann–Whitney U test or Kruskal–Wallis H test. Kaplan–Meier survival analysis was performed to analyze the survival of patients and gene expression. Luciferase assay and imaging analysis were performed at least twice to make sure that similar results could be reproduced. Experiments for CCK8, cell transwell assay, colony formation, RT-PCR, and immunoblotting experiments were repeated in A549 and H1975 cells. *p* < 0.05 was considered as statistically significant difference.

### Reporting summary

Further information on research design is available in the Nature Portfolio Reporting Summary linked to this article.

### Supplementary information

Supplementary Information

Description of Additional Supplementary Files

Supplementary Data 1

Reporting Summary

## Data Availability

All other data are available from the corresponding author (or other sources, as applicable) on reasonable request. The numerical source data behind the graphs in the manuscript can be found in Supplementary Data 1. The Uncropped Gels were shown Supplementary Fig. 11.
