# Supplementary material for: m6A-methylated KCTD21-AS1 regulates macrophage phagocytosis through CD47 and cell autophagy through TIPR
Source: Commun Biol. 2024 Feb 21;7:215. doi: 10.1038/s42003-024-05854-x (PMC10881998; doi:10.1038/s42003-024-05854-x)

Supplemental Data

Supplemental Table 1. The demographic and clinicopathological characteristics of patients with NSCLC

	Patients/n (%)
**Total**	20
**Gender**	
**Male**	8 (40.0)
**Female**	12 (60.0)
**Age(mean±SD,Years)**	59.15±14.554
**Pathological type**	
**Adenocarcinomas**	42 (56.0)
**Squamous cell carcinomas**	24 (32.0)
**Small cell lung cancer**	9 (12.0)
**TNM stage**	
Ⅰ-Ⅱ	20 (26.7)
Ⅲ-Ⅳ	48 (64.0)
**Unclear**	7 (9.3)
**lymph node metastasis**	
**Yes**	48 (64.0)
**No**	16 (21.3)
**Unclear**	11 (14.7)

Supplemental Table 2. The primers used to amplify genes in this study

Genes	Sequences (5’ to 3’)
TIPRL-Forward	GAAAGCAGGACGGAGGGTGAACA
Reverse	AAACCGCAACAGCAGGAAAAAGC
METTL14-Forward	GTAGCACAGACGGGGACTTC
Reverse	GAGCCAGCCTGGTCGAATTG
KCTD21-AS1-Forward	GCCACTCGTACTTGCTGTCTC
Reverse	TTTGGTTCTTCTTTCCTCTCC
miR-519d-5p	CCTCCAAAGGGAAGCGCTTTCTGTT
5S rRNA	GCCATACCACCCTGAACG
RTQ	AACATGTACAGTCCATGGATG
GAPDH-Forward	GACAGTCAGCCGCATCTTCTT
Reverse	AATCCGTTGACTCCGACCTTC
TIPRL-3′-UTR-Forward	CGCGAATTCCTGTCTTCACATTCATATTCCAG
Reverse	CGGCTCGAGATCCTGATTTCCTATAGCTTGGA
CD47-3′-UTR-Forward	CGCGAATTCAATAACTGAAGTGAAGTGATG
Reverse	CGGCTCGAG ATCACCAGGGCAGTGCTAAG

Supplemental figures and figure legends

Supplemental Figure 1: KCTD21-AS1 expression analysis and overexpression and siRNA vectors.

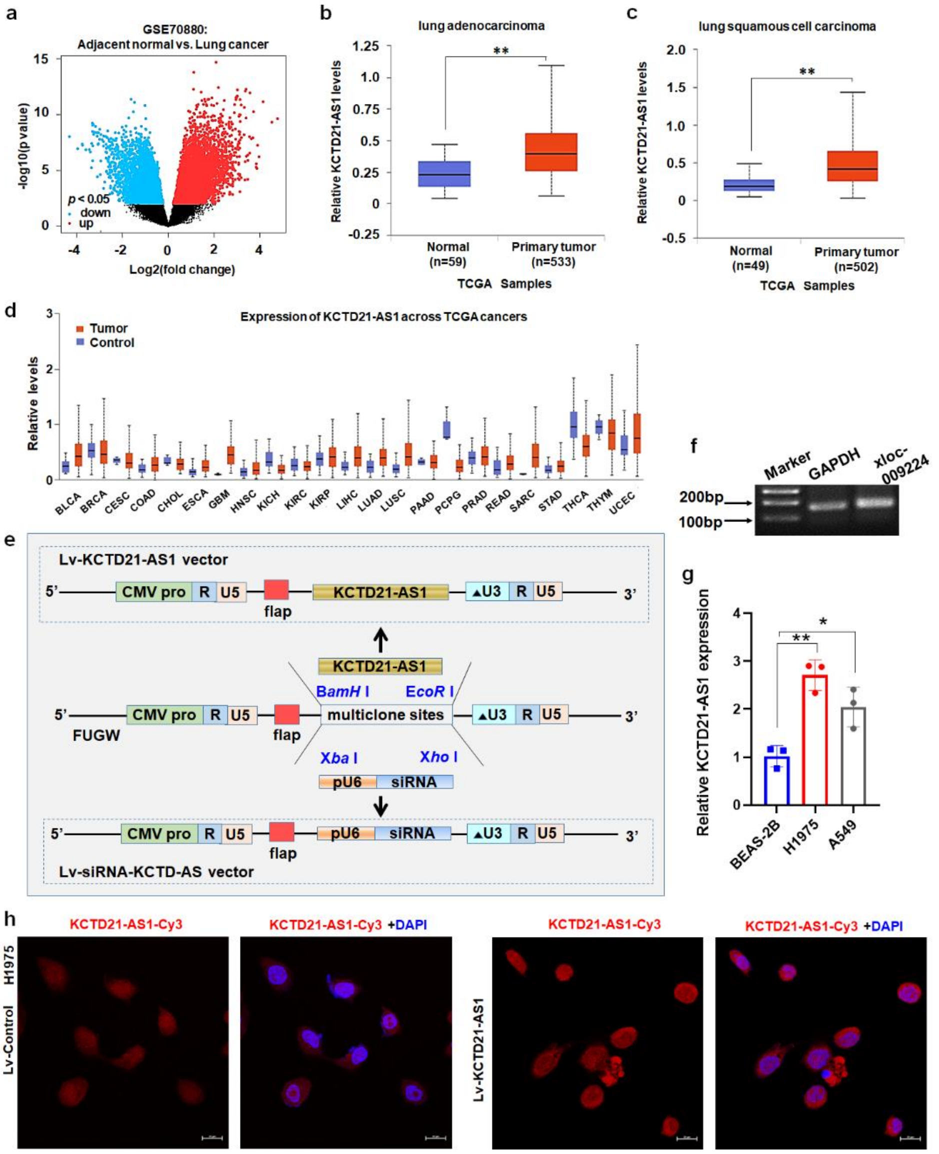

a Volcano plot of GSE70880 lncRNA microarray datasets.

b,c KCTD21-AS1 expression was higher in lung adenocarcinoma and lung squamous cell carcinoma tissues than in controls (data from TCGA). $^{**}p < 0.01$.

d The data from TCGA indicated that KCTD21-AS1 is increased in many cancers.

e Structure of KCTD21-AS1 overexpression and siRNA vectors.

siRNA sequence: 5'-CCGTAACGTGAATCTGCA-3'.

f RT-PCR showed that the detection of KCTD21-AS1 expression in lung cancer cells.

g KCTD21-AS1 expression was detected in H1975, A549, and BEAS-2B cells. *$p <$ 0.05, **$p < 0.01$, ANOVA.

h *In situ* hybridization detection of KCTD21-AS1 in H1975. The red color indicates KCTD21-AS1 expression. Bar = 20 μm.

Supplemental Figure 2: KCTD21-AS1 promoted cell proliferation in H1975 cells

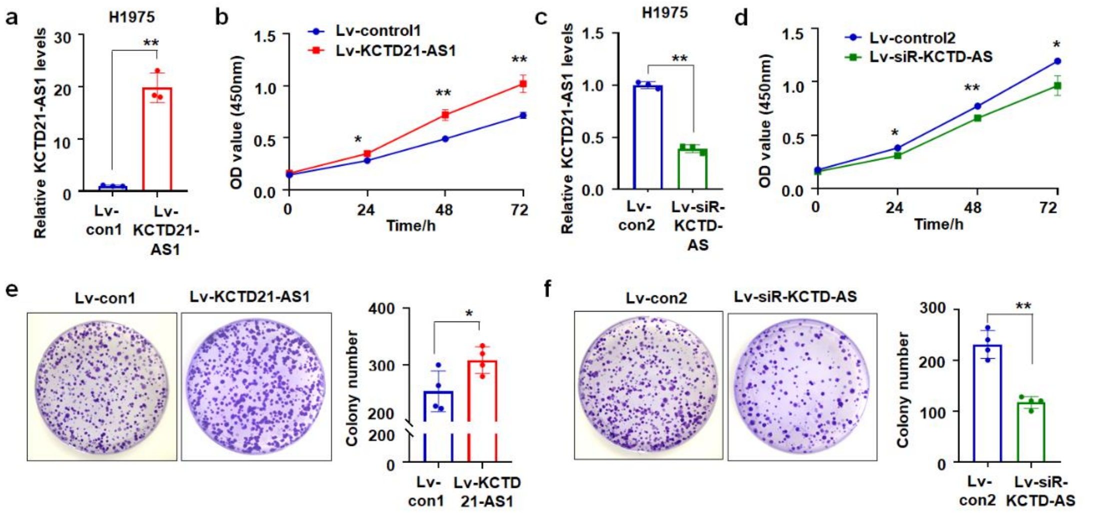

a,b KCTD21-AS1 overexpression promoted H1975 cell growth.

c,d siRNA-KCTD21-AS1 inhibited H1975 cell proliferation.

e,f KCTD21-AS1 overexpression promoted, siRNA-KCTD21 inhibited H1975 cell colony formation.

Data are presented as the mean ± SD for triplicate experiments. *$p < 0.05$; **$p < 0.01$; Student's t-test.

Supplemental Figure 3: The expression of KCTD21-AS1-related factors in xenografts

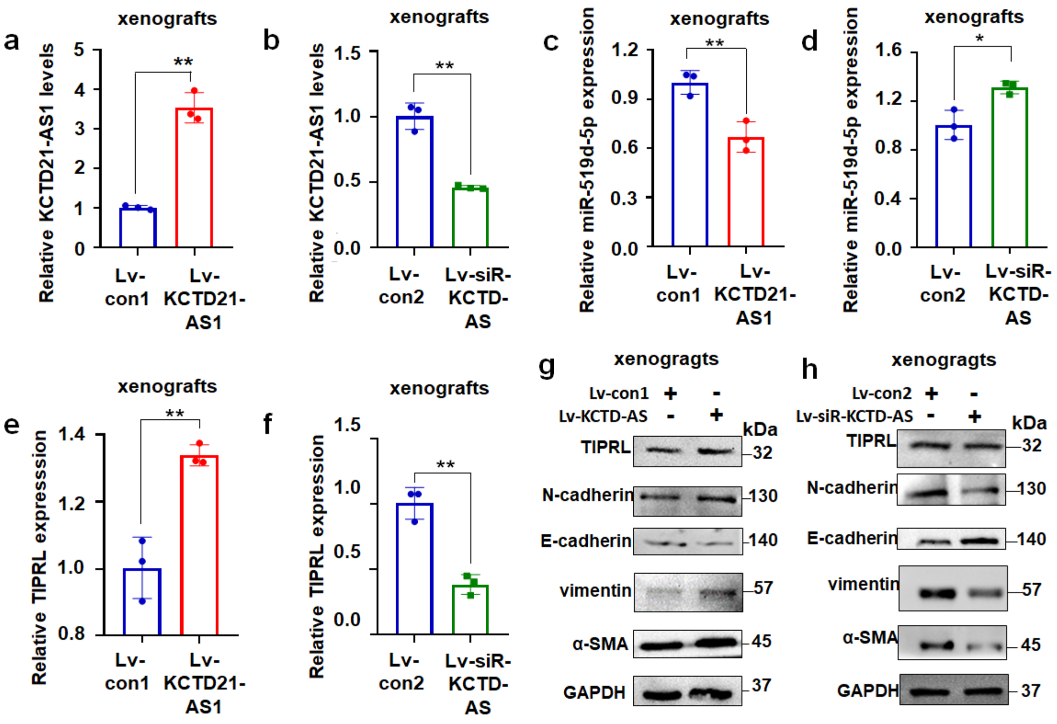

a,b KCTD21-AS1 expression in lv-KCTD21-AS1 and lv-siRNA-KCTD21-AS -treated xenografts.

c,d miR-519d-5p levels in lv-KCTD21-AS1 and lv-siRNA-KCTD21-AS-treated xenografts.

e,f TIPRL levels in lv-KCTD21-AS1 and lv-siRNA-KCTD21-AS-treated xenografts.

g,h E-cadherin, N-cadherin, Vimentin, and α-SMA were detected by immunoblotting from different membrane, and the sample loading amount according to the reference control, which was run on a different gel than the corresponding sample of interest.

Data are presented as the mean ± SD for triplicate experiments. *$p < 0.05$; **$p < 0.01$; Student's t-test.

Supplemental Figure 4: miRNAs regulated luciferase levels in A549 cells.

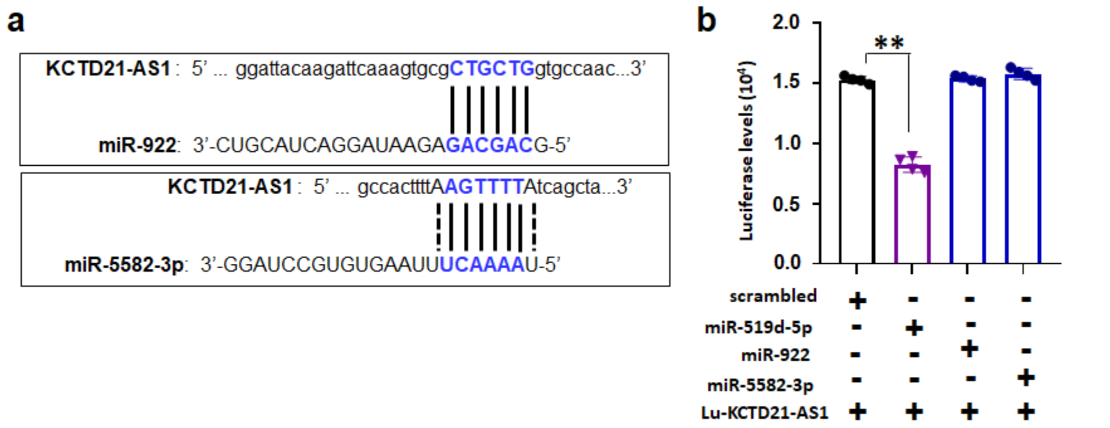

a the interaction sites between miRNAs (miR-922 and miR-5582-3p) and KCTD21-AS1;

b the pc3.1-luci-KCTD21 vector with the binding sites of miR-519d-5p, miR-922, and miR-5582-3p was constructed to investigated the interaction between KCTD21-AS1 and miRNAs. The role of miRNAs in regulating luciferase levels in pc3.1-luci-KCTD21-treated A549 cells showed that the luciferase levels decreased in the miR-519d-5p-treated cells, but not in miR-922- or miR-5582-3p-treated cells.

Data are presented as the mean ± SD for triplicate experiments. $^{**}p < 0.01$; ANOVA.

Supplemental Figure 5: miR-519d-5p inhibited H1975 cell growth.

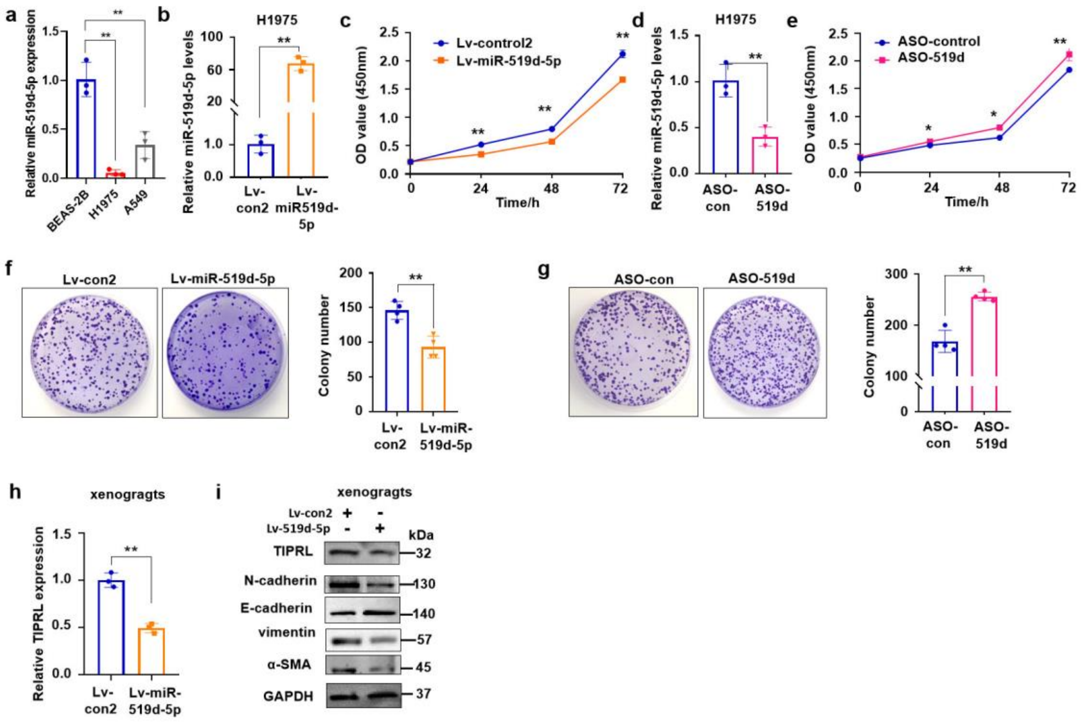

a miR-519d-5p expression in NSCLC cells.

b,c miR-519d-5p upregulation inhibited H1975 cell proliferation.

d,e miR-519d-5p inhibitor promoted H1975 cell proliferation.

f,g miR-519d-5p decreased, whereas ASO-519d-5p increased H1975 cell colony formation.

h TIPRL expression in miR-519d-5p-treated xenografts.

i E-cadherin, N-cadherin, Vimentin, and α-SMA were analyzed by immunoblotting from different membrane, and the sample loading amount according to the reference control, which was run on a different gel than the corresponding sample of interest.

Data are presented as the mean ± SD for triplicate experiments. *$p < 0.05$; **$p < 0.01$; Student's t-test.

Supplemental Figure 6: miR-519d-5p recovery prevented KCTD21-AS1-promoting cell proliferation.

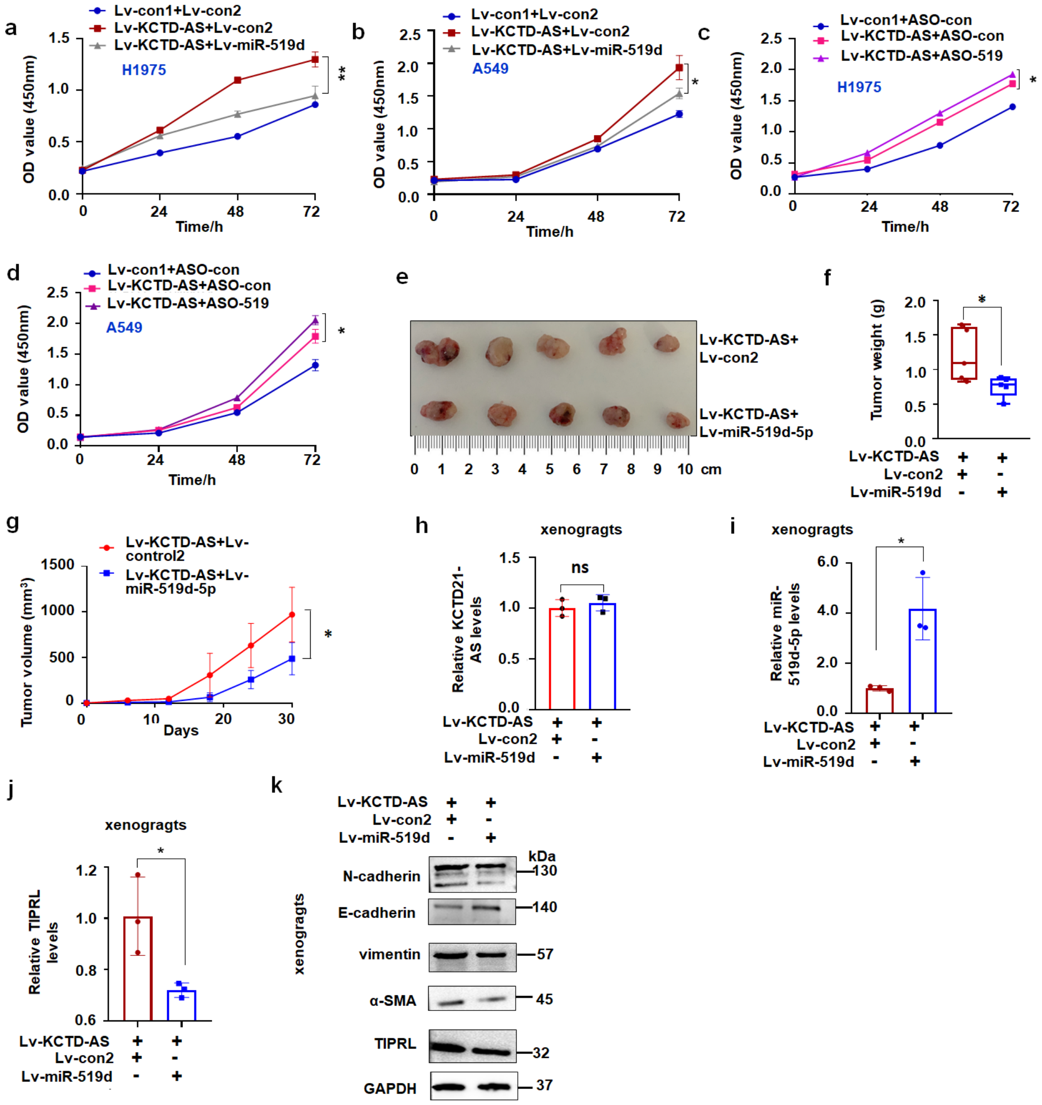

a,b Effect of miR-519d-5p on the role of KCTD21-AS1-promoting cell proliferation.

c,d Effect of ASO-miR-519d on the role of KCTD21-AS1-promoting cell proliferation.

e-g Tumor size, weight, and volume changes were measured in lv-KCTD21-AS1+lv-miR-519d-treated xenografts, n=5.

h-j The expression of KCTD21-AS1, miR-519d-5p and TIPRL was detected in xenografts, respectively.

k E-cadherin, N-cadherin, Vimentin, TIPRL and α-SMA were analyzed by immunoblotting from different membrane, and the sample loading amount according

to the reference control, which was run on a different gel than the corresponding sample of interest.

Data are presented as the mean ± SD or median (interquartile range). *$p < 0.05$; **$p < 0.01$; Student's t-test, ANOVA, or Mann–Whitney U test.

Supplemental Figure 7: The role of miR-519d-5p and KCTD21-AS1 in cell metastasis.

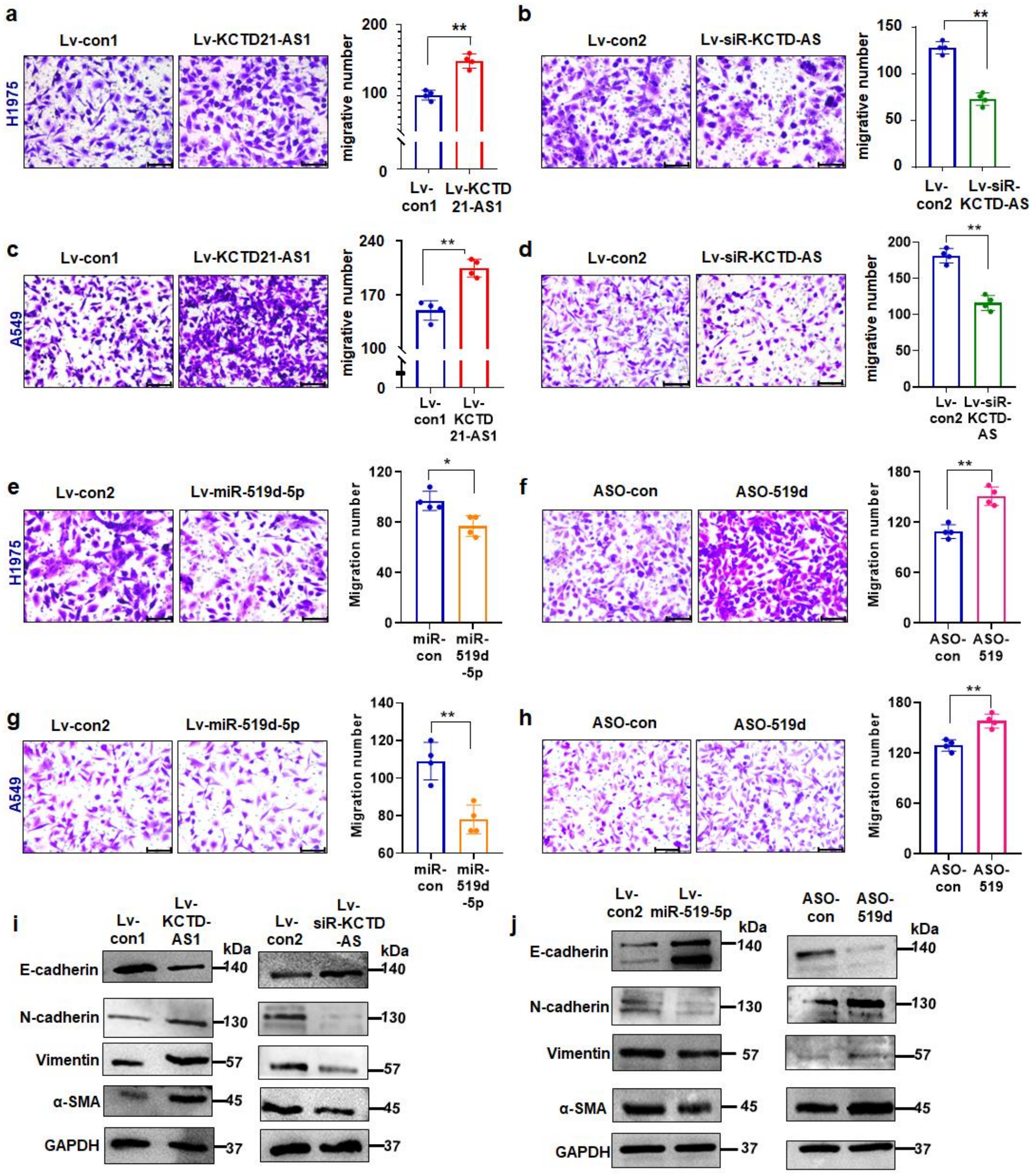

a,b KCTD21-AS1 upregulation promoted and siRNA-KCTD21-AS1 suppressed H1975 cell metastasis. Bar = 100 μm.

c,d Efect of KCTD21-AS1 upregulation and siRNA-KCTD21-AS1 on A549 cell

metastasis. Bar = 100 µm.

e,f miR-519d-5p inhibited and ASO-519d promoted H1975 cell metastasis. Bar = 100 µm.

g,h The effect of miR-519d-5p and ASO-519d on A549 cell metastasis. Bar = 100 µm.

i E-cadherin, N-cadherin, Vimentin, and α-SMA were analyzed in KCTD21-AS1- and siRNA-treated cells from different membrane, and the sample loading amount according to the reference control, which was run on a different gel than the corresponding sample of interest.

j E-cadherin, N-cadherin, Vimentin, and α-SMA were analyzed in miR-519d- and ASO-treated cells from different membrane, and the sample loading amount according to the reference control, which was run on a different gel than the corresponding sample of interest.

Data are presented as the mean ± SD for triplicate experiments. *$p < 0.05$; **$p < 0.01$; Student's t-test.

Supplemental Figure 8. The role of miR-519d-5p in affecting KCTD21-AS1-promoting cell metastasis

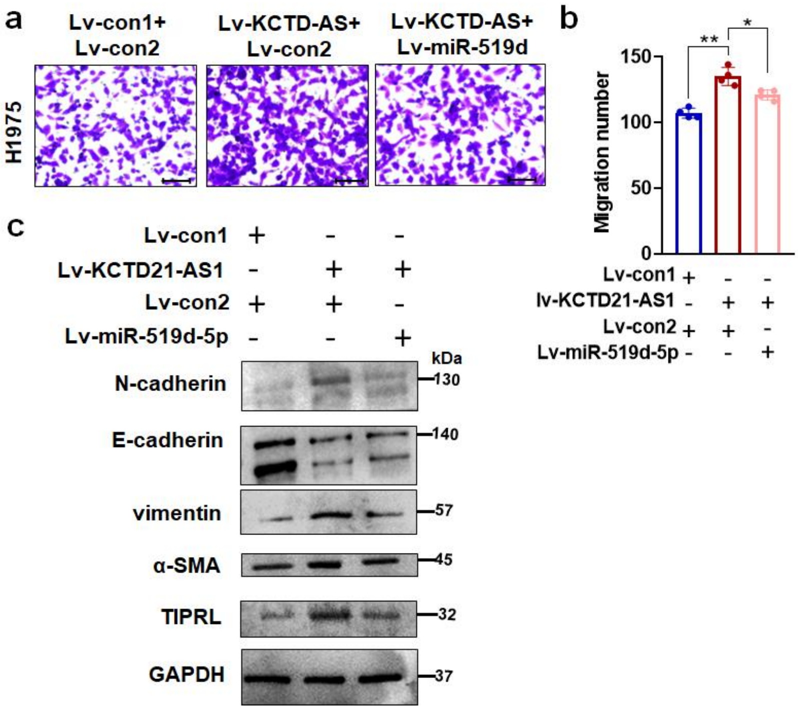

a,b Effect of miR-519d-5p on affecting KCTD21-AS1-promoting H1975 cell metastasis. Bar = 100 μm.

c E-cadherin, N-cadherin, Vimentin, TIPRL and α-SMA were analyzed from different membrane, and the sample loading amount according to the reference control, which was run on a different gel than the corresponding sample of interest.

Data are presented as the mean ± SD for triplicate experiments. **p* < 0.05; ***p* < 0.01; ANOVA.

Supplemental Figure 9: The negative control lung tissues and HE-staining.

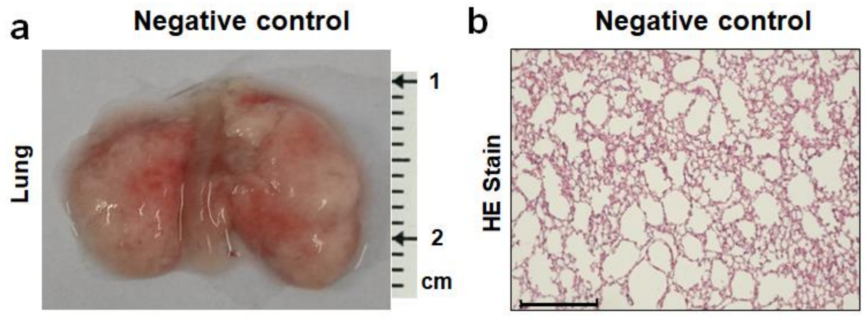

a Visualization of lung tissues.

b The HE-stained lung section. Bar=125 μm.

Supplemental Figure 10: The role of CD-47 in affecting miR-519d-regulating cell metastasis.

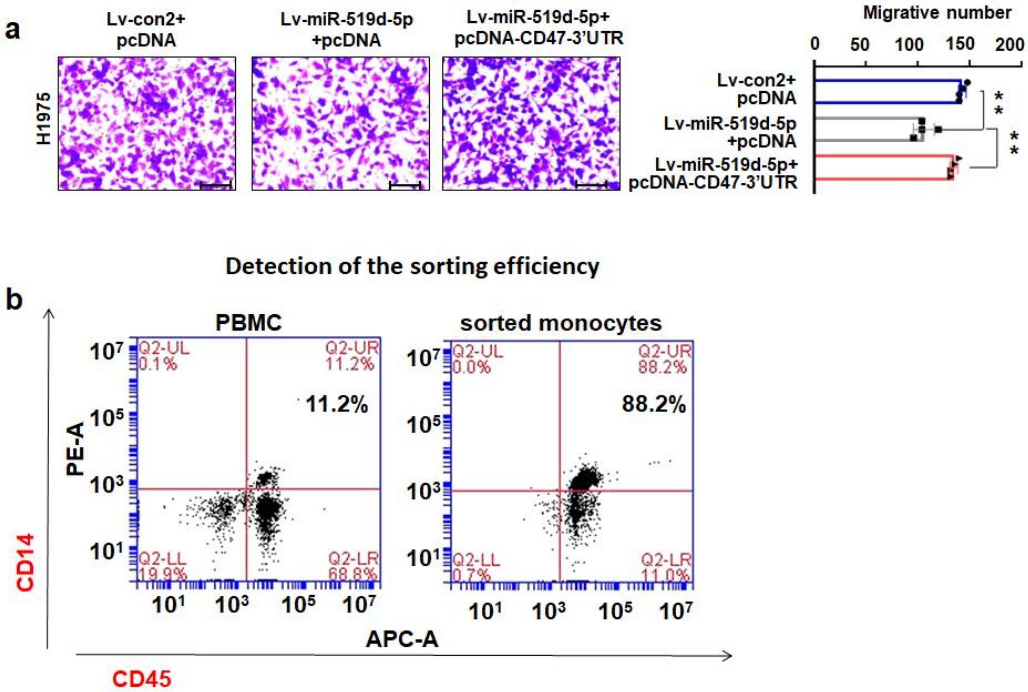

a CD47-3′UTR recovery attenuated miR-519d-5p-suppressing cell migration. Data are presented as the mean ± SD for triplicate experiments. Bar = 100 μm. $^{**}p < 0.01$; ANOVA.

b The macrophages were sorted and co-cultured in vitro. $CD45^{+}$cells were selecting using the APC fluorescent channel gate, and then $CD45^{+}CD14^{+}$ monocytes were selected using the PE fluorescent channel.

Supplemental Figure 11:

Uncropped gels

Figure 3a

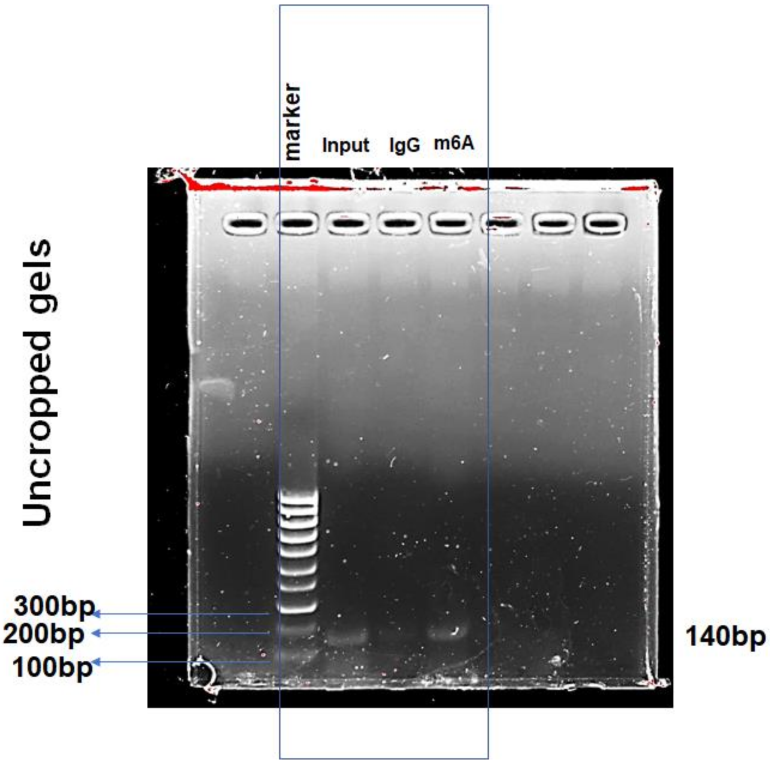
Uncropped gels
marker
Input
IgG
m6A
300bp
200bp
100bp
140bp

Figure 4d

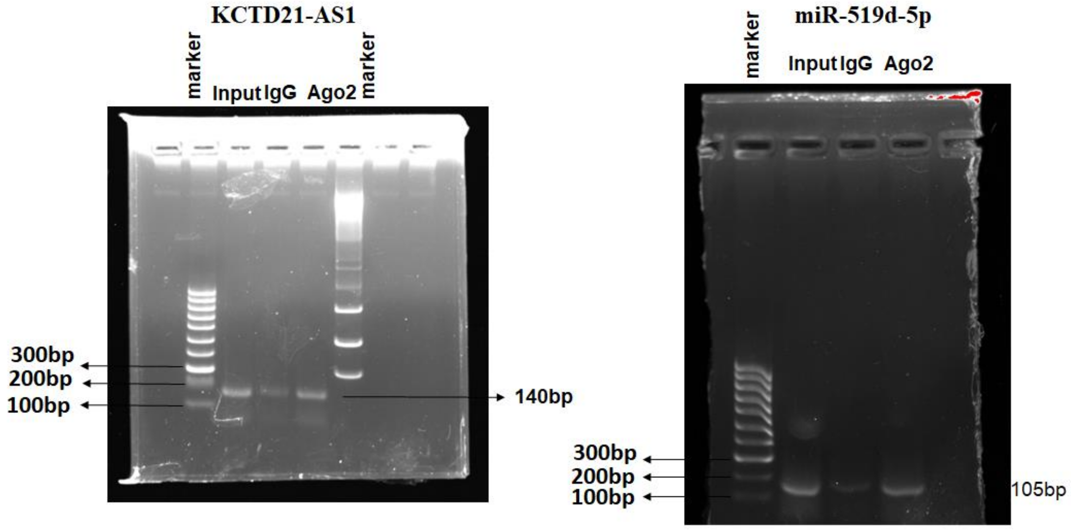
KCTD21-AS1
marker
Input
IgG
Ago2
marker
300bp
200bp
100bp
140bp
miR-519d-5p
marker
Input
IgG
Ago2
300bp
200bp
100bp
105bp

Figure 6

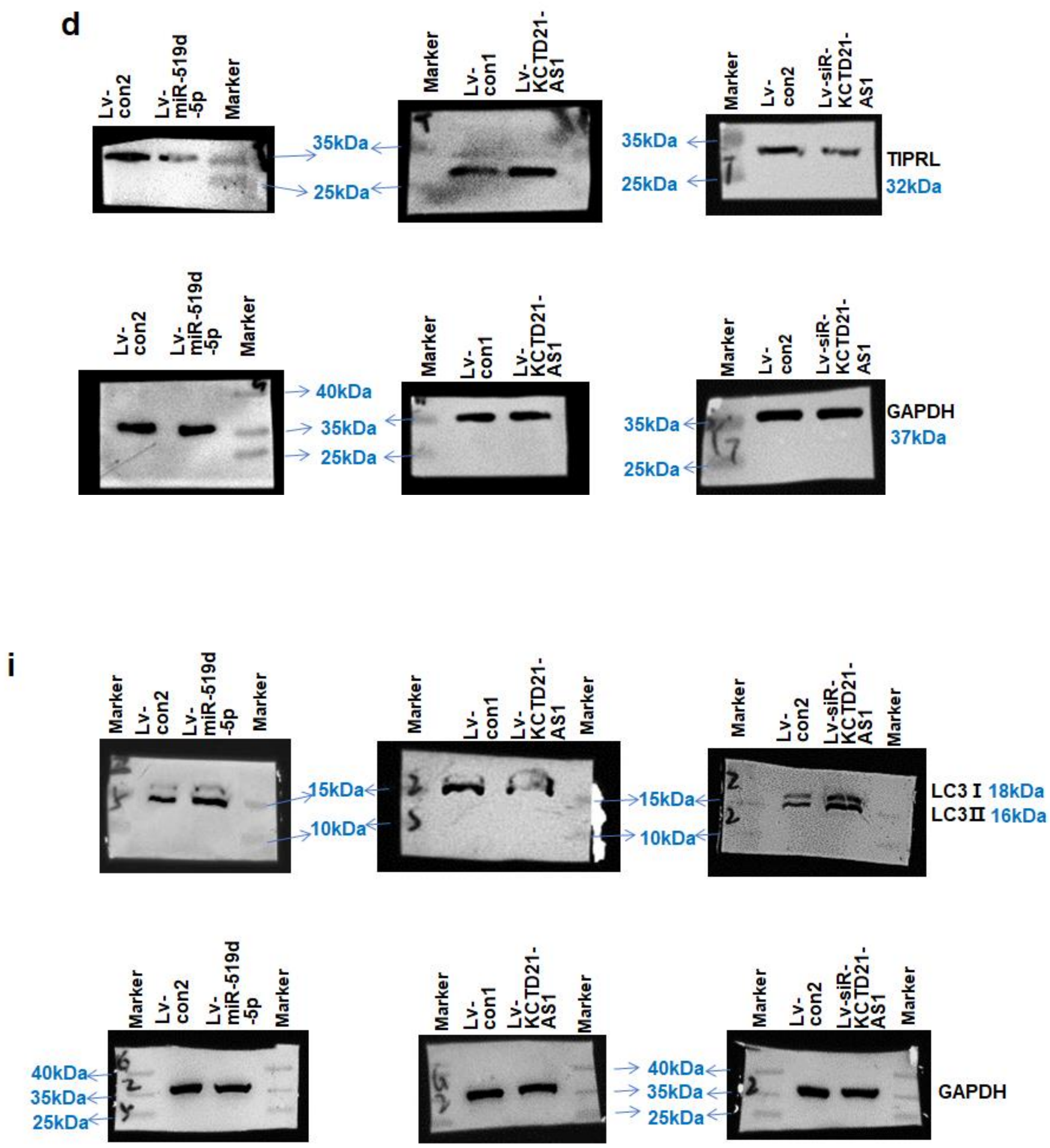
d
Lv-con2
Lv-miR-519d-5p
Marker
Lv-con1
Lv-KCTD21-AS1
Lv-siR-KCTD21-AS1
35kDa
25kDa
40kDa
TIPRL
32kDa
GAPDH
37kDa
i
15kDa
10kDa
LC3 I 18kDa
LC3 II 16kDa

Figure 7c

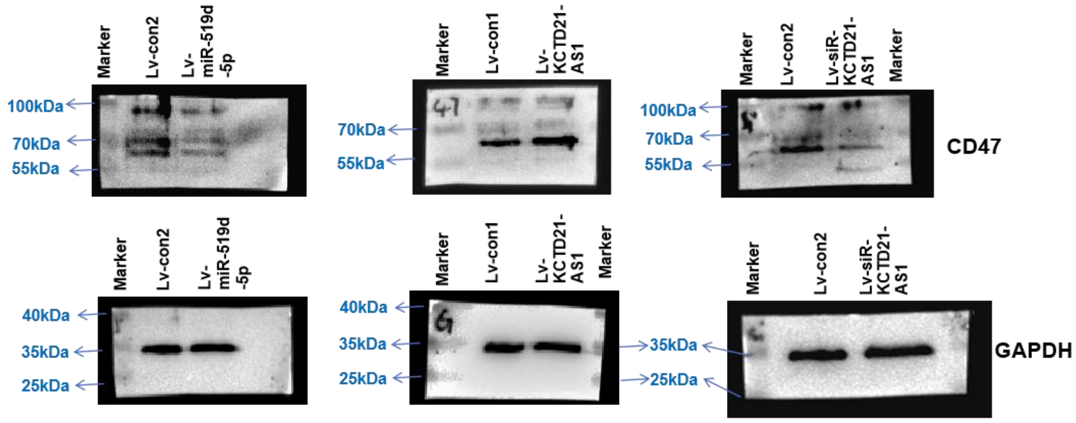
Marker
Lv-con2
Lv-miR-519d-5p
Lv-con1
Lv-KCTD21-AS1
Lv-siR-KCTD21-AS1
100kDa
70kDa
55kDa
40kDa
35kDa
25kDa
CD47
GAPDH

Supplemental Figure 1f

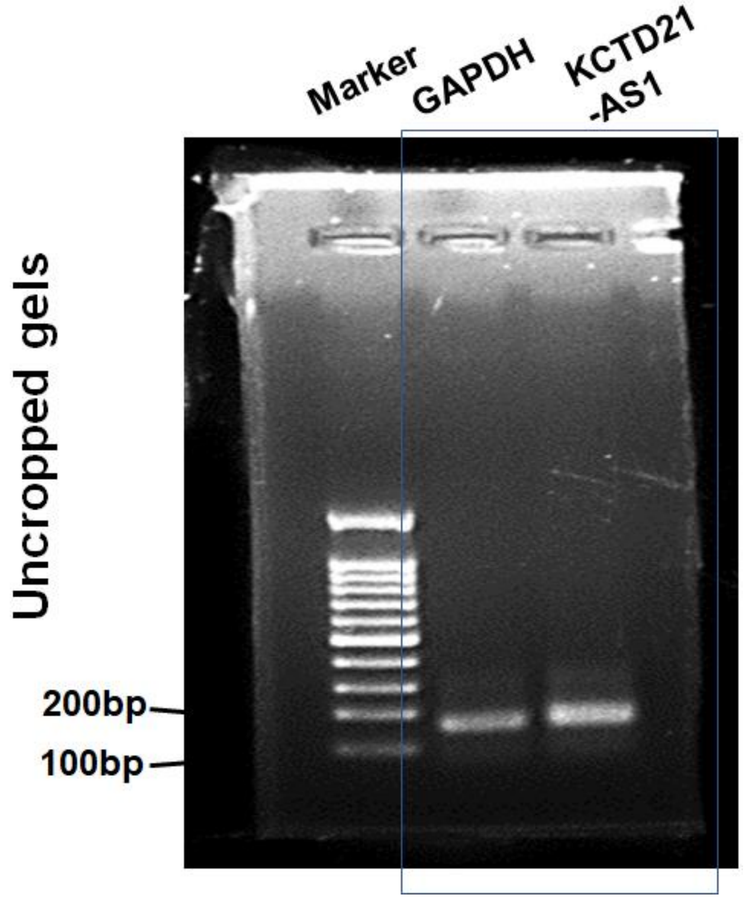

Supplemental Figure 3g,h

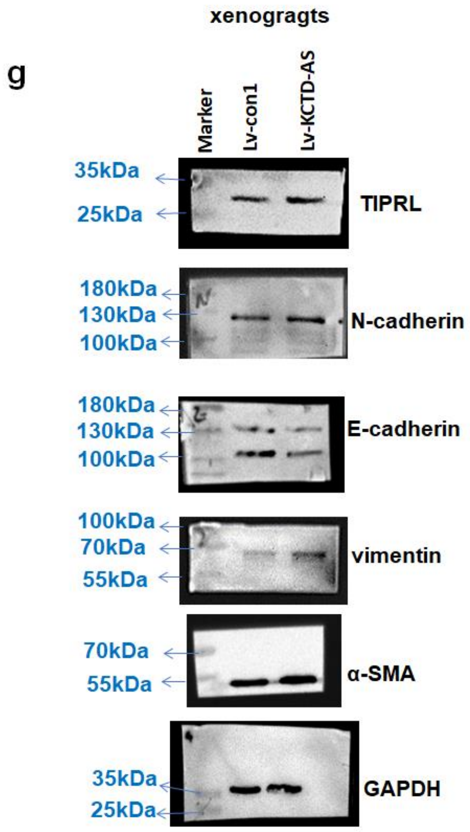

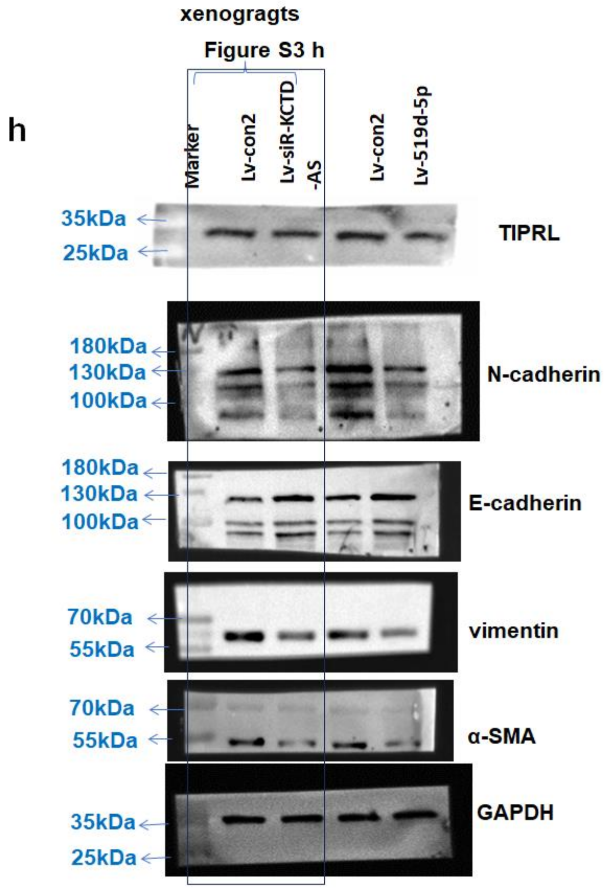

Supplemental Figure 5i

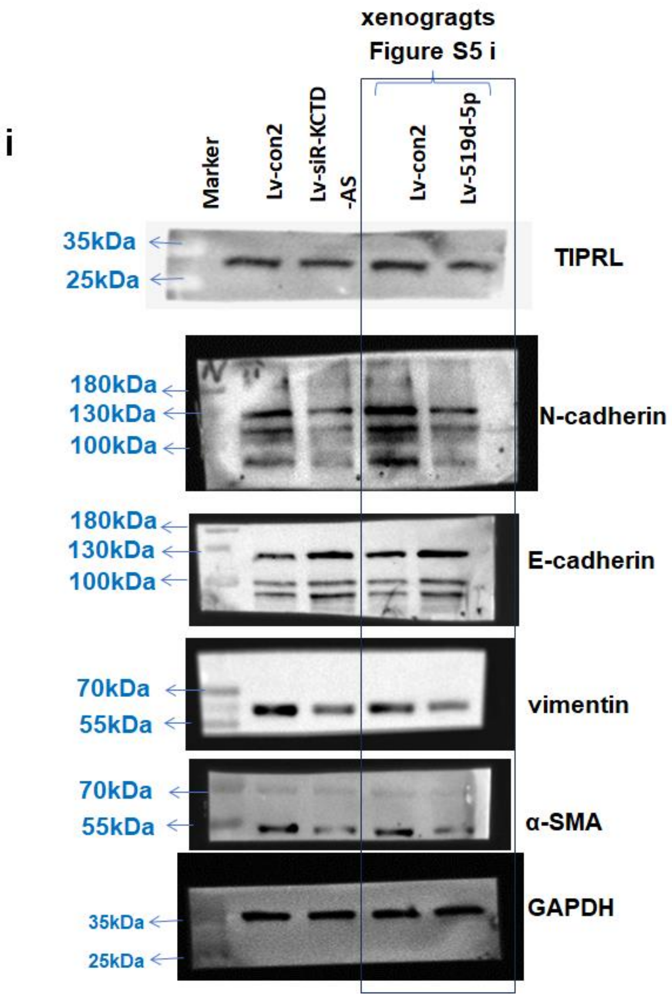

Supplemental Figure 6k

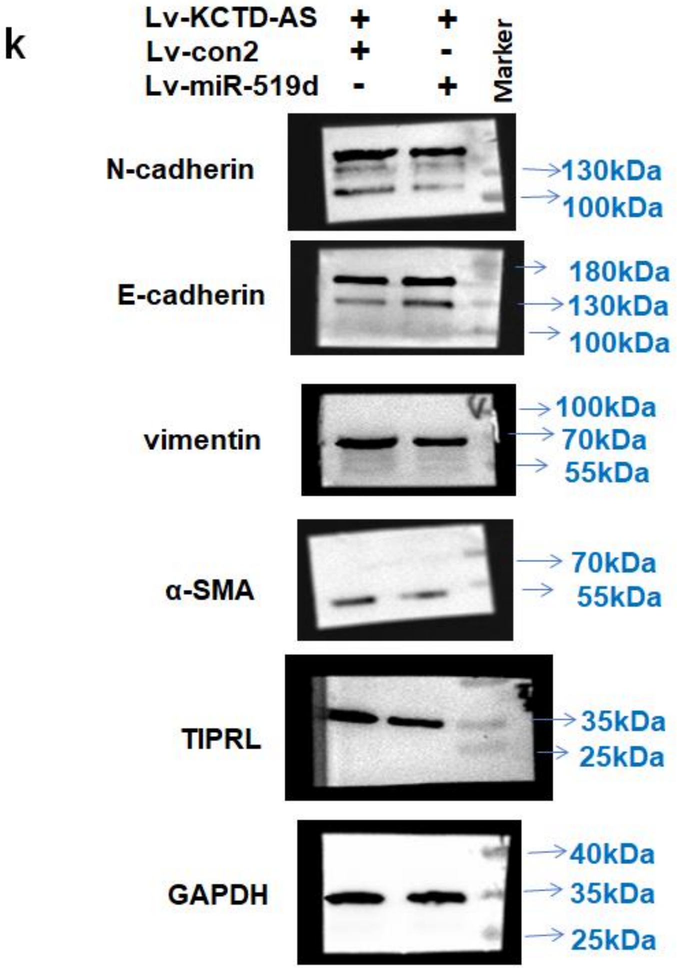

Supplemental Figure 7i,j

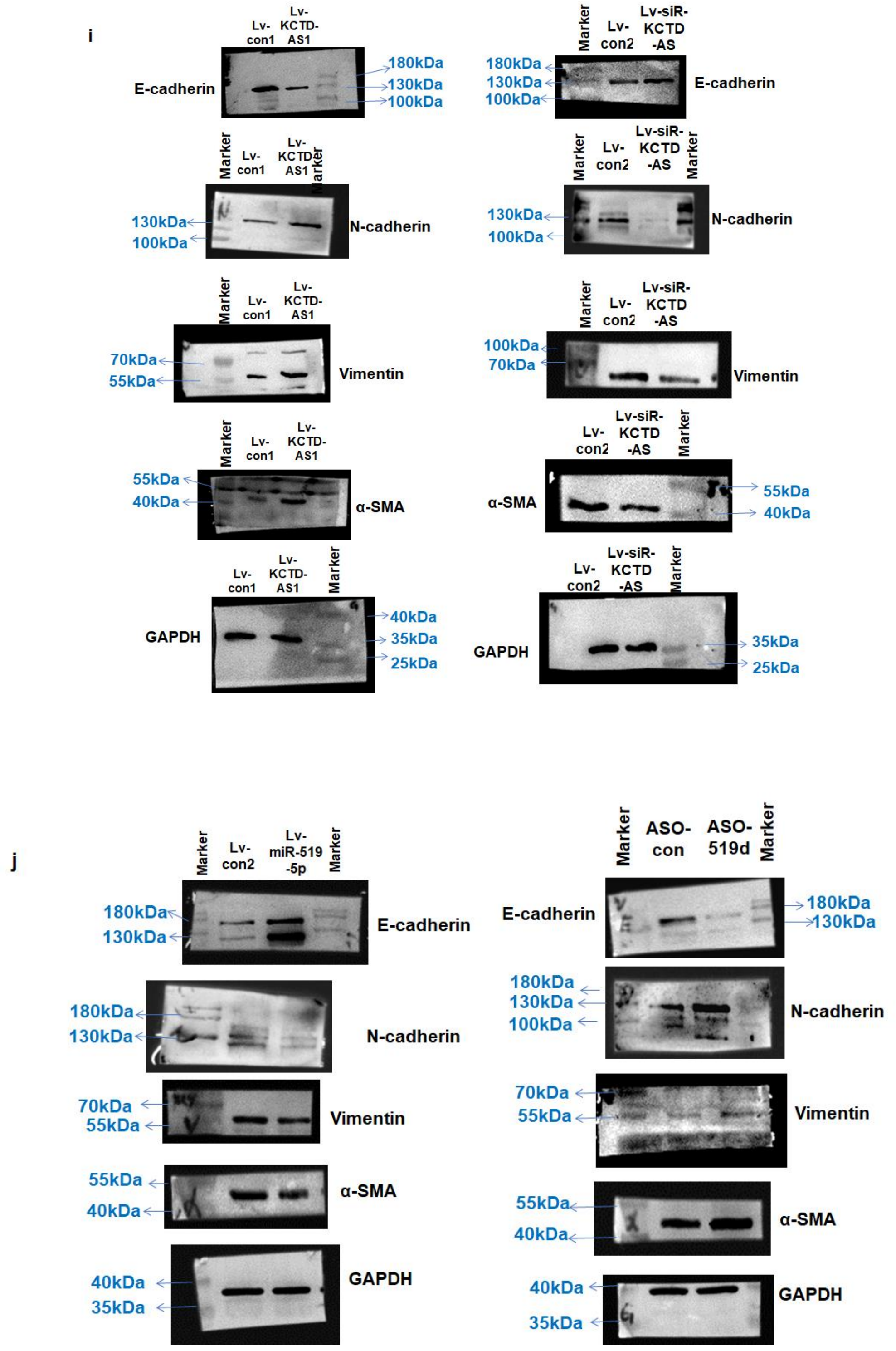

Supplemental Figure 8c

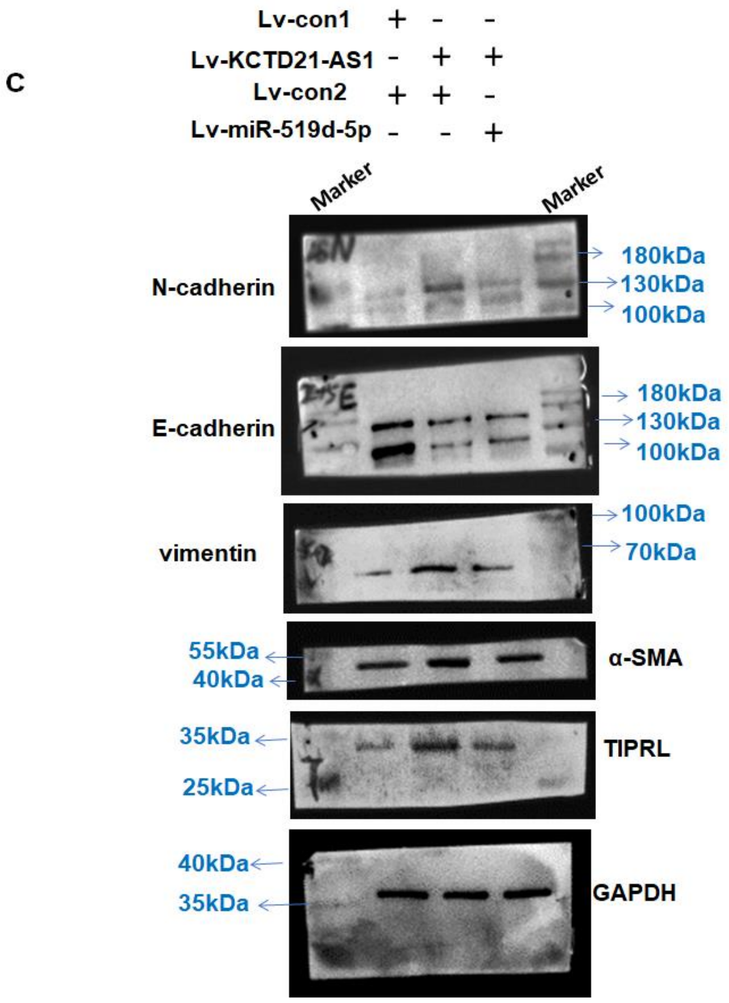

Supplement: Supplementary file 1 — Supplementary Information [file 42003_2024_5854_MOESM1_ESM.pdf]
