# Supplementary material for: m6A-methylated KCTD21-AS1 regulates macrophage phagocytosis through CD47 and cell autophagy through TIPR
Source: Commun Biol. 2024 Feb 21;7:215. doi: 10.1038/s42003-024-05854-x (PMC10881998; doi:10.1038/s42003-024-05854-x)
Supplement: Supplementary file 2 — Description of Additional Supplementary Files [file 42003_2024_5854_MOESM2_ESM.pdf]

Description of Additional Supplementary Files

File name: Supplementary Data 1

Description: The numerical source data for graphs in the manuscript.
