# Supplementary material for: m6A-methylated KCTD21-AS1 regulates macrophage phagocytosis through CD47 and cell autophagy through TIPR
Source: Commun Biol. 2024 Feb 21;7:215. doi: 10.1038/s42003-024-05854-x (PMC10881998; doi:10.1038/s42003-024-05854-x)
Supplement: Supplementary file 4 — Reporting Summary [file 42003_2024_5854_MOESM4_ESM.pdf]

nature portfolio

Corresponding author(s): Shu-Yang Xie

Last updated by author(s): Nov 28, 2023

Reporting Summary

Nature Portfolio wishes to improve the reproducibility of the work that we publish. This form provides structure for consistency and transparency in reporting. For further information on Nature Portfolio policies, see our Editorial Policies and the Editorial Policy Checklist.

Statistics

For all statistical analyses, confirm that the following items are present in the figure legend, table legend, main text, or Methods section.

n/a	Confirmed	
☐	☒	The exact sample size (*n*) for each experimental group/condition, given as a discrete number and unit of measurement
☐	☒	A statement on whether measurements were taken from distinct samples or whether the same sample was measured repeatedly
☐	☒	The statistical test(s) used AND whether they are one- or two-sided<br>*Only common tests should be described solely by name; describe more complex techniques in the Methods section.*
☒	☐	A description of all covariates tested
☒	☐	A description of any assumptions or corrections, such as tests of normality and adjustment for multiple comparisons
☐	☒	A full description of the statistical parameters including central tendency (e.g. means) or other basic estimates (e.g. regression coefficient) AND variation (e.g. standard deviation) or associated estimates of uncertainty (e.g. confidence intervals)
☐	☒	For null hypothesis testing, the test statistic (e.g. *F*, *t*, *r*) with confidence intervals, effect sizes, degrees of freedom and *P* value noted<br>*Give P values as exact values whenever suitable.*
☒	☐	For Bayesian analysis, information on the choice of priors and Markov chain Monte Carlo settings
☒	☐	For hierarchical and complex designs, identification of the appropriate level for tests and full reporting of outcomes
☐	☒	Estimates of effect sizes (e.g. Cohen's *d*, Pearson's *r*), indicating how they were calculated

Our web collection on statistics for biologists contains articles on many of the points above.

Software and code

Policy information about availability of computer code

Data collection	For immunohistochemistry (IHC) analysis: an EVOSTM M7000 Imaging System (Thermo Fisher Scientific, USA).<br>For Immunofluorescence: examined under a confocal microscope with LAS AF (LEICA TCS SPE, Leica, Dresden, Germany).<br>For Luciferase assay: a chemiluminescence analyzer (Infinite 200 PRE, Tecan Austria GmbH).
Data analysis	All graphing and associated statistical analysis: Prism 8.0.1 (GraphPad), or SPSS 22.0 software.

For manuscripts utilizing custom algorithms or software that are central to the research but not yet described in published literature, software must be made available to editors and reviewers. We strongly encourage code deposition in a community repository (e.g. GitHub). See the Nature Portfolio guidelines for submitting code & software for further information.

Data

Policy information about availability of data

All manuscripts must include a data availability statement. This statement should provide the following information, where applicable:

- Accession codes, unique identifiers, or web links for publicly available datasets
- A description of any restrictions on data availability
- For clinical datasets or third party data, please ensure that the statement adheres to our policy

Provided in the manuscript, including GSE70880 datasets from GEO (http://www.ncbi.nlm.nih.gov/geo/) .

Research involving human participants, their data, or biological material

Policy information about studies with human participants or human data. See also policy information about sex, gender (identity/presentation), and sexual orientation and race, ethnicity and racism.

Reporting on sex and gender	Total 20<br>Gender<br>Male 8<br>Female 12<br>Age(mean±SD,Years)<br>59.15±14.554
Reporting on race, ethnicity, or other socially relevant groupings	Asian
Population characteristics	Patients with NSCLC.
Recruitment	Prior to inclusion in the study, patients were fully informed of the study procedure and signed a written informed consent.
Ethics oversight	This study was approved by the Binzhou Medical College Ethics Committee.

Note that full information on the approval of the study protocol must also be provided in the manuscript.

Field-specific reporting

Please select the one below that is the best fit for your research. If you are not sure, read the appropriate sections before making your selection.

☒ Life sciences ☐ Behavioural & social sciences ☐ Ecological, evolutionary & environmental sciences

For a reference copy of the document with all sections, see nature.com/documents/nr-reporting-summary-flat.pdf

Life sciences study design

All studies must disclose on these points even when the disclosure is negative.

Sample size	five in each group
Data exclusions	No data were excluded.
Replication	All data was reliably reproduced, which were stated in the Methods and figure legends. For example, data were expressed for triplicate experiments.
Randomization	The experiments that involve BALB/C-nu nude mice were randomized. All data analyses were based on objectively measurable data.
Blinding	The experiments that involve BALB/C-nu nude mice were blinded. The experimental data were analyzed based on objectively measurable data. Blinding was not performed for in vitro study, because the researchers needed to know the treatments to conduct experiments, and the data were analyzed based on objectively measurable data.

Reporting for specific materials, systems and methods

We require information from authors about some types of materials, experimental systems and methods used in many studies. Here, indicate whether each material, system or method listed is relevant to your study. If you are not sure if a list item applies to your research, read the appropriate section before selecting a response.

Materials & experimental systems

n/a	Involved in the study	
☐	☒	Antibodies
☐	☒	Eukaryotic cell lines
☒	☐	Palaeontology and archaeology
☐	☒	Animals and other organisms
☐	☒	Clinical data
☒	☐	Dual use research of concern
☒	☐	Plants

Methods

n/a	Involved in the study	
☒	☐	ChIP-seq
☐	☒	Flow cytometry
☒	☐	MRI-based neuroimaging

Antibodies

Antibodies used	mouse anti-human E-cadherin (1:500, 20874-1-AP, proteintech, USA); rabbit anti-human N-cadherin (1:500, 22018-1-AP, proteintech); rabbit anti-human TIPRL (1:5000, AB70795, Abcam, MO, USA); rabbit anti-human α-SMA (1:500, BS70000, Biowworld, MN, USA); rabbit anti-human vimentin (1:500,BS1855, Biowworld); rabbit anti-human METTL14 (1:500, 31591, SAB, Nanjing, China); rabbit anti-human CD47 (1:500, A00360-2, Boster); rabbit anti-human LC3B (1:500, BM4827, Boster); and rabbit anti-human GAPDH (1:3000, AP0063); and mouse anti-human GAPDH (1:3000, MB001) (both from Biowworld Technology).
Validation	All antibodies have been validated by their manufacturers; the manufacturer's websites stating the validation (including the species and application) of each antibody were listed above. The dilution/final concentration of each of the antibodies is determined experimentally by us.

Eukaryotic cell lines

Policy information about cell lines and Sex and Gender in Research

Cell line source(s)	The human BEAS-2B, A549, H1975, and 293T cell lines were obtained from the Shanghai Institute of Cell Biology.
Authentication	BEAS-2B, A549, H1975, and 293T cells were verified by PCR genotyping.
Mycoplasma contamination	All cell lines were tested negative for mycoplasma contamination.
Commonly misidentified lines (See ICLAC register)	No commonly misidentified lines were used.

Animals and other research organisms

Policy information about studies involving animals; ARRIVE guidelines recommended for reporting animal research, and Sex and Gender in Research

Laboratory animals	BALB/C-nu nude mice were from HFK Bio-Technology, Beijing, China;
Wild animals	N/A
Reporting on sex	Female BALB/C-nu nude mice
Field-collected samples	No field-collected samples were employed.
Ethics oversight	All animal experiments were performed in accordance with the Guidelines for Care and Use of Laboratory Animals of National Institutes of Health guidelines and approved by the Committee on the Ethics of Animal Experiments of Binzhou Medical University.

Note that full information on the approval of the study protocol must also be provided in the manuscript.

Clinical data

Policy information about clinical studies

All manuscripts should comply with the ICMJE guidelines for publication of clinical research and a completed CONSORT checklist must be included with all submissions.

Clinical trial registration	N/A
Study protocol	N/A
Data collection	N/A
Outcomes	N/A

Plants

Seed stocks: N/A

Novel plant genotypes: N/A

Authentication: N/A

Flow Cytometry

Plots

Confirm that:

☒ The axis labels state the marker and fluorochrome used (e.g. CD4-FITC).

☒ The axis scales are clearly visible. Include numbers along axes only for bottom left plot of group (a 'group' is an analysis of identical markers).

☒ All plots are contour plots with outliers or pseudocolor plots.

☒ A numerical value for number of cells or percentage (with statistics) is provided.

Methodology

Sample preparation: N/A

Instrument: N/A

Software: N/A

Cell population abundance: N/A

Gating strategy: N/A

☒ Tick this box to confirm that a figure exemplifying the gating strategy is provided in the Supplementary Information.
